# Cell wall protection by the *Candida albicans* class I chitin synthases

**DOI:** 10.1016/j.fgb.2015.08.001

**Published:** 2015-09

**Authors:** Kanya Preechasuth, Jeffrey C. Anderson, Scott C. Peck, Alistair J.P. Brown, Neil A.R. Gow, Megan D. Lenardon

**Affiliations:** a Aberdeen Fungal Group, School of Medical Sciences, Institute of Medical Sciences, University of Aberdeen, Foresterhill, Aberdeen AB25 2ZD, United Kingdom; b Division of Biochemistry, 271H Bond Life Sciences Center, University of Missouri-Columbia, Columbia, MO 65211, USA

**Keywords:** YFP, yellow fluorescent protein, CFW, Calcofluor White, S222, serine at position 222, MIC, minimal inhibitory concentration, rpm, revolutions per minute, FCS, foetal calf serum, 5-FOA, 5-fluoroorotic acid, LB, Luria-Bertani, bp, base pairs, PBS, phosphate buffered saline, PI, propidium iodide, SEM, standard error of the mean, ANOVA, analysis of variance, SD, standard deviation, *Candida albicans*, Cell wall, Chitin synthase, Class I chitin synthase, Chs2, Chs8, Polarized growth, Phosphorylation

## Abstract

*Candida albicans* has four chitin synthases from three different enzyme classes which deposit chitin in the cell wall, including at the polarized tips of growing buds and hyphae, and sites of septation. The two class I enzymes, Chs2 and Chs8, are responsible for most of the measurable chitin synthase activity *in vitro*, but their precise biological functions *in vivo* remain obscure. In this work, detailed phenotypic analyses of a *chs2*Δ*chs8*Δ mutant have shown that *C. albicans* class I chitin synthases promote cell integrity during early polarized growth in yeast and hyphal cells. This was supported by live cell imaging of YFP-tagged versions of the class I chitin synthases which revealed that Chs2-YFP was localized at sites of polarized growth. Furthermore, a unique and dynamic pattern of localization of the class I enzymes at septa of yeast and hyphae was revealed. Phosphorylation of Chs2 on the serine at position 222 was shown to regulate the amount of Chs2 that is localized to sites of polarized growth and septation. Independently from this post-translational modification, specific cell wall stresses were also shown to regulate the amount of Chs2 that localizes to specific sites in cells, and this was linked to the ability of the class I enzymes to reinforce cell wall integrity during early polarized growth in the presence of these stresses.

## Highlights

• Class I chitin synthases promote cell integrity during early polarized growth.
• Class I enzymes show a unique and dynamic pattern of localization at septa.
• Phosphorylation of Chs2 regulates the amount of protein localized to septation sites.
• Cell wall stresses independently regulate the amount of Chs2 at specific sites.
• Class I enzymes provide protection from cell wall stresses in early polarized growth.

## 1 Introduction

Chitin is an essential structural polysaccharide component of cell walls and septa in fungi and is synthesized by multiple chitin synthase enzymes. Importantly, chitin is not found in human cells and therefore represents an attractive target for antifungal therapy. In *Candida albicans*, the most common serious fungal pathogen of humans, chitin is synthesized by a family of four isoenzymes which fall into three different classes of chitin synthase enzymes, Chs1 (class II), Chs3 (class IV), Chs2 and Chs8 (class I) (reviewed in Lenardon et al. (2010b)). Together, these enzymes deposit chitin at sites of growth, which includes the polarized tips of buds and hyphae, and sites of septation. Understanding how these enzymes coordinately synthesize chitin in fungal cells is a vitally important aspect of fungal cell biology and will inform strategies to exploit chitin as a target for the development of new antifungal drugs. The present study shows for the first time that the *C. albicans* class I enzymes contribute to the protection of the nascent cell wall during polarized growth and the integrity of cells experiencing cell wall stress.

Analysis of *chs* mutant phenotypes has given us clues about the individual roles of the chitin synthases during growth and cell division. For example, Chs1 is essential and is responsible for the synthesis of the primary septum (Munro et al., 2001), while Chs3 synthesizes the majority of chitin found in the cell wall as well as the chitin ring at division sites (Bulawa et al., 1995). The localization of Chs1-YFP and Chs3-YFP in live cells has provided further evidence to support these roles for Chs1 and Chs3 (Lenardon et al., 2007). The role of the two class I enzymes (Chs2 and Chs8) is less well understood, and these are revealed here.

Previous work has shown that deletion of *CHS2* and *CHS8* results in a 97–99% reduction of the chitin synthase activity that can be measured *in vitro*, with the deletion of *CHS2* alone accounting for an 80–91% reduction compared to wild-type (Munro et al., 2003), but *chs2*, *chs8* or *chs2chs8* mutants display few other obvious phenotypes under normal growth conditions (Gow et al., 1994; Mio et al., 1996; Munro et al., 2003). The expression profile of the class I genes indicates that they may be involved in providing protection to cells during cell wall stresses since *CHS2* and *CHS8* are 3–3.5-fold up-regulated at the level of transcription when cells are grown in the presence of caspofungin, an echinocandin drug which targets β(1,3)-glucan synthesis in fungal cell walls (Walker et al., 2008), and 9–12-fold up-regulated when cells are grown in the presence of CaCl_2_ and Calcofluor White (CFW) (Munro et al., 2007). This up-regulation of transcription correlates with an overall increase in the *in vitro* chitin synthase activity in membranes prepared from yeast cells treated with caspofungin or CaCl_2_ and CFW (Munro et al., 2007; Walker et al., 2008). More recently, it has been shown that Chs2, and Chs2 and Chs8 can form salvage septa in the absence of all other chitin synthases, including the normally essential Chs1, provided that chitin synthesis has been activated by pre-treatment of cells with CaCl_2_ and CFW (Walker et al., 2013). It is also known that the effect of the Chs1 inhibitor (RO-09-3143) on wild-type cells is fungistatic, whereas it is fungicidal in a *chs2* mutant background (Sudoh et al., 2000). These observations suggest that Chs2 and Chs8 have significant biological functions under stress conditions that are not yet fully understood.

Other studies have shown that Chs8 is involved in chitin microfibril morphogenesis. *CHS8* is required for the synthesis of long chitin microfibrils in the septa of yeast and hyphae, and Chs2-YFP and Chs8-YFP are both located at sites of septation in yeast cells immediately prior to cytokinesis (Lenardon et al., 2007). Chs8-YFP has also been observed at septation sites in hyphae (Lenardon et al., 2007). A global analysis of the *C. albicans* phosphoproteome showed that Chs2 is phosphorylated on the serine at position 222 (S222) (Beltrao et al., 2009), although the significance of the phosphorylation of class I chitin synthases has not been investigated. Ultimately, the true biological function of the class I enzymes in *C. albicans* remains to be clarified.

The objective of this work was therefore to elucidate the biological function of Chs2 and Chs8 during normal growth conditions in *C. albicans*, to investigate the biological consequence of phosphorylation of Chs2 on S222 and to investigate the mechanism(s) by which the class I enzymes provide protection to cells during cell wall stress.

## 2 Materials and methods

### 2.1 Strains, media and growth conditions

Yeast cells were grown in YPD broth (1% (w/v) yeast extract, 2% (w/v) mycological peptone, 2% (w/v) glucose supplemented with 25 μg/ml uridine (YPD+uri), or SD broth (0.67% (w/v) yeast nitrogen base without amino acids with ammonium sulphate, 2% (w/v) glucose supplemented with 25 μg/ml uridine at 30 °C with shaking at 200 rpm. In some experiments, YPD+uri broth was supplemented with a sub-MIC concentration of caspofungin (0.016 μg/ml Cancidas®, Merck and Co., Inc., USA), or 0.2 M CaCl_2_ and 0.1 mg/ml CFW (Fluorescent Brightener 28, Sigma–Aldrich Co.). To induce hyphal growth, yeast cells that had been grown overnight in YPD+uri were inoculated into pre-warmed 20% (v/v) foetal calf serum (FCS) supplemented with 25 μg/ml uridine at a concentration of 1 × 10^7^ cells/ml and incubated at 37 °C with shaking at 200 rpm. Transformants were selected and maintained on SD plates (SD broth with 2% (w/v) purified agar) with appropriate auxotrophic supplements, and recycling of the *URA3* marker was achieved by plating cells on SD plates containing 0.1% (w/v) 5-fluoroorotic acid (5-FOA) and uridine (25 μg/ml). *E. coli* strains containing the plasmids pBSCHS2-2, pDDB57 and pYFP-URA3 were grown on Luria-Bertani (LB) medium (0.5% (w/v) yeast extract, 1% (w/v) tryptone, and 1% (w/v) NaCl) supplemented with 100 μg/ml ampicillin. *E. coli* containing pSN52 was grown on LB medium supplemented with 30 μg/ml kanamycin.

### 2.2 Construction of chs mutant strains

A heterozygous *CHS8*/*chs8* mutant was constructed using a PCR based method adapted from Noble and Johnson (2005). Primers MDL24 and MDL25 (Table S1) with 100 bp homology to the sequence immediately upstream of the start codon and 100 bp immediately downstream of the stop codon of *CHS8* were designed to anneal to sequences immediately adjacent to the *Candida dubliniensis HIS1* marker in pSN52 (Noble and Johnson, 2005). The resulting PCR product was transformed into *C. albicans* strain BWP17 (Table 1). His^+^ colonies were screened by PCR using primers MDL28 and MDL29 (Table S1) to confirm that one copy of *CHS8* had been replaced by the *CdHIS1* marker. The resulting strain was designated *CHS8*/*chs8*Δ*0* (NGY609, Table 1).

The second *CHS2* and *CHS8* allele of the *CHS2*/*chs2*Δ*0* and *CHS8*/*chs8*Δ*0* heterozygous mutants (NGY479 and NGY609; Table 1) was disrupted using the mini ura-blaster method (Wilson et al., 2000). The disruption cassette containing *dpl200-URA3-dpl200* flanked by 100 bp of sequence homologous to the region of *CHS2* or *CHS8* immediately 5′ to the start codon and 100 bp of sequence homologous to the region of *CHS2* or *CHS8* immediately 3′ of the stop codon was PCR-amplified from pDDB57 (Wilson et al., 2000) using primers KP08, KP09 or KP17, KP18 (Table S1). The PCR product was transformed into the *CHS2*/*chs2*Δ*0* or *CHS8*/*chs8*Δ*0* heterozygous mutant. Ura^+^ colonies were screened by PCR using primers KP10 and KP11 or KP19 and KP11 (Table S1). After 5-FOA selection to recycle *URA3*, the correct construction of the strains was confirmed by PCR using primers KP10 and KP12 or KP19 and KP20 (Table S1) and by Southern analysis using a probe generated by PCR using the primers KP13 and KP14 or KP21 and KP22 (Table S1) which amplifies the sequence from −511 to −29 relative to ATG*^CHS2^* or −808 to −220 relative to ATG*^CHS8^*. These strains were designated *chs2*Δ*0* and *chs8*Δ*0* (NGY603 and NGY608; Table 1).

*C. albicans* strains that express mutant forms of Chs2 were constructed by re-introducing one copy of the mutated *chs2* alleles at the native chromosomal locus of the *chs2*Δ*0* null mutant strain (NGY603; Table 1). The promoter, ORF and terminator of the *CHS2* gene (−664 to +3177 relative to ATG*^CHS2^*) were amplified by PCR using the primers CHS2Ia and CHS2IIb (Table S1) which introduced an *Apa*I and *Sal*I site at each end. Similarly, the *CHS2* terminator (+3031 to +3177 relative to ATG*^CHS2^*) was amplified using the primers CHS2IIIa and Chs2IIIb (Table S1) which introduced an *Xba*I and *Not*I site at each end. The two PCR products were then ligated between the *Apa*I and *Sal*I sites, and the *Xba*I and *Not*I sites respectively of pBS-URA3 (Lenardon et al., 2010a) to generate the plasmid pBSCHS2-2. pBSCHS2-2 contains the *CHS2* promoter, *CHS2* ORF, *CHS2* terminator, *URA3* and a second copy of the *CHS2* terminator between the *Not*I and *Apa*I sites in pBluescript SK+ (Stratagene) which facilitates the integration of the *Not*I-*Apa*I cassette at the *CHS2* locus by homologous recombination and recycling of the *URA3* selective marker.

The QuickChange®II XL Site-Directed Mutagenesis kit (Stratagene) was used to generate site-specific mutations in the *CHS2* ORF in the plasmid pBSCHS2-2. An S222A mutation was generated by changing the TCA at position +664 in the *CHS2* ORF to GCA using the mutagenic oligonucleotide primers KPS222A1 and KPS222A2 (Table S1). This point mutation created a version of Chs2 that cannot be phosphorylated on S222. To generate a form of Chs2 that mimics constitutive phosphorylation on S222, an S222E mutation was introduced by changing the TCA to GAA using the mutagenic oligonucleotide primers KPS222E1 and KPS222E2 (Table S1). Point mutations were confirmed by DNA sequencing before further manipulations. The mutated plasmids were designated pBSCHS2-2(S222A) and pBSCHS2-2(S222E).

Plasmids pBSCHS2-2(S222A) and pBSCHS2-2(S222E) were digested with *Not*I and *Apa*I to generate cassettes that were transformed into the *chs2*Δ*0* null mutant strain (NGY603; Table 1). Ura^+^ transformants were screened by PCR using the primers KP15 and KP07 (Table S1). Ura^−^ colonies were selected on 5-FOA plates and were screened by PCR using primers KP16 and KP12 (Table S1) and then confirmed by Southern analysis. The introduction of the targeted mutations was confirmed by DNA sequencing. These strains were designated *chs2*^S222A^ and *chs2*^S222E^ (NGY604 and NGY605; Table 1). The *in vitro* chitin synthase activity of these strains was measured using a radiolabeled substrate incorporation assay as described in Munro et al. (1998).

### 2.3 Construction of YFP-tagged strains

Strains expressing a C-terminally YFP-tagged version of Chs8, Chs2^S222A^ and Chs2^S222E^ were constructed by fusing *YFP* to the 3′ end of the remaining *CHS8* allele in *CHS8*/*chs8*Δ*0* (NGY609; Table 1) or of the phospho-mutant alleles of *CHS2* in *chs2*^S222A^ and *chs2*^S222E^ (NGY604 and NGY605; Table 1) using the method described in Lenardon et al. (2007). Briefly, primers MDL7 and MDL8 or MDL3 and MDL4 (Table S1) were used to amplify a cassette from pYFP-URA3 (Gerami-Nejad et al., 2001) containing *YFP*, the *ADH1* terminator and *URA3* flanked by 100 bp of sequence homologous to the regions immediately upstream and downstream of the stop codon of *CHS8* or *CHS2*. This cassette was transformed into *CHS8*/*chs8*Δ*0* (NGY609; Table 1) or *chs2*^S222A^ and *chs2*^S222E^ (NGY604 and NGY605; Table 1). Ura^+^ colonies were screened using primers MDL14 and MDL16R or MDL12 and MDL16R (Table S1) and the correct integration of the YFP cassette was confirmed by Southern analysis. The resulting strains were designated *CHS8-YFP*/*chs8*Δ*0* (NGY602), *chs2*^S222A^-YFP (NGY606) and *chs2*^S222E^-YFP (NGY607) (Table 1). The expression of the YFP-tagged versions of Chs8 and Chs2 in these strains was confirmed by western blot using the method described in Lenardon et al. (2007).

### 2.4 Microscopy

To acquire single snap-shot images of yeast cells in single focal plane, overnight cultures of strains were diluted 1:100 into fresh YPD+uri or SD broth and incubated at 30 °C with shaking at 200 rpm. Cells were washed and resuspended in phosphate buffered saline (PBS), mounted on a glass slide, sealed under a cover slip and examined under a DeltaVision Core microscope (Applied Precision). Counterstaining of cells with CFW or propidium iodide (PI) was achieved by adding 10 μg/ml CFW or 2 μg/ml PI to the washed cells as they were mounted on the glass slide. Images were acquired with a CoolSNAP camera (Photometrics). Time-lapse movies of budding yeast cells and growing hyphae were made by growing cells on agar pads as described previously (Lenardon et al., 2010a; Veses and Gow, 2008) using a DeltaVision Core microscope (Applied Precision) with a QuantEM:512SC camera (Photometrics). Where appropriate, 10 μg/ml CFW, 2 μg/ml PI, 0.016 μg/ml (sub-MIC) or 0.032 μg/ml (near-MIC) caspofungin, or 0.2 mM CaCl_2_ and 0.1 mg/ml CFW were also added into the agar pads. YFP, CFW and PI fluorescence were detected using a standard FITC, DAPI and Rh-TRITC filter set, respectively (Chroma Technology Corporation).

For quantification of the fluorescence intensity of single YFP spots at septa, the intensity of the spot was measured using ImageJ (http://rsbweb.nih.gov/ij/). The circle selection tool was used to select and measure the “mean gray value” for the area immediately surrounding the single YFP spot, as well as for an area of background. The “mean gray value” for the background in each image was subtracted from the “mean gray value” for each spot. At least 20 spots were measured for each strain/condition and the results presented as the average of these intensity measurements (±SEM).

### 2.5 Statistical analyses

Contingency tables (Chi-squared test) were used to determine whether there was an overall effect on the distribution of the frequencies of PI-positive phenotypes resulting from the deletion of the class I genes (i.e. wild-type compared to the *chs2*Δ*chs8*Δ mutant strain) or treatment with caspofungin, as opposed to occurring due to chance alone. Subsequent Mann–Whitney *U* tests were carried out in IBM® SPSS® Statistics 22. For these analyses, the percentages of cells displaying individual PI-positive phenotypes analyzed were those obtained from the three independent biological replicates, and not simply the totals which are represented in Tables 3 and 4. IBM® SPSS® Statistics 20 was used the comparison of YFP fluorescence intensities. Multiple classes of data were compared by ANOVA, and pair-wise comparisons by Student’s *t*-test. In all cases, *P* values ⩽ 0.05 were deemed statistically significant.

## 3 Results

### 3.1 Class I chitin synthases are important for cell integrity during early polarized growth in yeast and hyphal cells

**Fig. 1:**
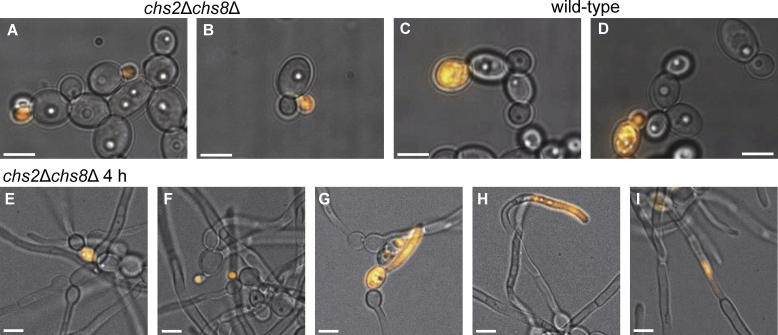
Phenotypic analyses of *chs2*Δ*chs8*Δ mutant yeast and hyphae. A–D. Yeast cells of the *chs2*Δ*chs8*Δ mutant and wild-type (CAI-4) strains were grown in SD broth at 30 °C for 24 h and counterstained with PI. Panels show representative images of PI-positive stained small buds in the *chs2*Δ*chs8*Δ mutant (A and B), PI-positive stained large buds in wild-type cells (C), and PI-positive stained mother-and-bud in wild-type cells (D). E–I. Hyphae of the *chs2*Δ*chs8*Δ mutant were grown on 20% FCS agar pads with PI at 37 °C for 4 h. Panels show representative images of the PI-positive phenotypes indicative of dead mother cells (E), dead buds (F), dead short hyphae (G), dead end compartments (H) and dead mid-compartments (I). All panels show the overlay of PI fluorescence on the corresponding DIC image. Scale bars represent 5 μm.

Our initial observations showed that cell lysis, determined by the staining of non-viable cells and cell compartments with the vital stain PI, was enhanced in certain cell wall mutant backgrounds. Therefore, we probed the biological function of the *C. albicans* class I chitin synthases by analyzing cell lysis phenotypes of the *chs2*Δ, *chs8*Δ and *chs2*Δ*chs8*Δ mutants using PI. Yeast cells were grown in SD broth for 24 h, counter-stained with PI, and imaged by fluorescence microscopy. Dead cells that could not exclude PI were observed in higher proportions in cultures of yeast cells of the *chs2*Δ and *chs2*Δ*chs8*Δ mutants (Table 1) compared to the wild-type strain (CAI-4; Table 1) (Table 2). The percentage of PI-positive cells of the *chs2*Δ*chs8*Δ double mutant was more than the sum of the percentages of PI-positive cells of the *chs2*Δ and *chs8*Δ mutants, and the deletion of *CHS2* resulted in a higher proportion of PI-positive cells than the deletion of *CHS8* (Table 2). Approximately 4% of small buds of the *chs2*Δ*chs8*Δ mutant were stained with PI indicating that death occurred during early polarized growth in these cells (Fig. 1A,B and Table 2). This phenotype was not observed in the wild-type strain (Table 2). However, there was no difference in the number of cells that stained PI-positive during later stages of the cell cycle, i.e. isotropic growth or just before cytokinesis (where the PI-positive stained bud was almost equal in size to that of the mother cell) in the wild-type and *chs2*Δ*chs8*Δ mutant (Fig. 1C and Table 2). The proportion of cells where both the mother-and-bud or unbudded mother cell stained PI-positive (Dead mother-and-bud or unbudded cells, Table 2) accounted for approximately 2% of *chs2*Δ*chs8*Δ mutant and 1% of wild-type yeast cells (Fig. 1D). These results suggest that together, Chs2 and Chs8 are important for maintaining cell integrity during early polarized growth in the yeast cell cycle, but less so in later phases of the cell cycle where growth occurs by isotropic cell expansion, or at cytokinesis.

This analysis was extended to hyphal cells by inducing hyphal growth on agar pads containing 20% FCS and PI at 37 °C for 4 h and 24 h and imaging cells by fluorescence microscopy. A number of different PI-positive phenotypes were observed in hyphae (Table 3 and Fig. 1E–I). These included PI-positive stained mother cells (Dead mothers, Table 3 and Fig. 1E), PI-positive stained buds (Dead buds, Table 3 and Fig. 1F), short germ tubes that had not yet laid down the first septum hyphal septum that were stained PI-positive (Dead short hyphae, Table 3 and Fig. 1G), hyphae where the apical compartment only was stained with PI (Dead end-compartments, Table 3 and Fig. 1H), and hyphae where one non-apical compartment was stained PI-positive (Dead mid-compartments, Table 3 and Fig. 1I). Cells that were not stained with PI were considered to be alive.

The distribution of the frequencies of these phenotypes that were observed in wild-type and *chs2*Δ*chs8*Δ mutant hyphae was compared. Over all, there was a significant difference between the wild-type and *chs2*Δ*chs8*Δ mutant at both 4 h (Chi-squared = 40.72, 6 degrees of freedom, *P* < 0.005) and 24 h (Chi-squared = 16.93, 6 degrees of freedom, *P* < 0.01). Further statistical analyses revealed that after 4 h, there were more dead short hyphae and hyphae with dead end compartments in the *chs2*Δ*chs8*Δ mutant compared to wild-type (Table 3), and after 24 h, there were more dead-short hyphae in the *chs2*Δ*chs8*Δ mutant compared to wild-type. These results suggest that Chs2 and Chs8 play a role in maintaining cell integrity during early polarized hyphal growth, and immediately following septation events in hyphae.

### 3.2 Chs2-YFP is localized to sites of polarized growth and Chs2-YFP and Chs8-YFP show similar patterns of localization at septation sites in yeast and hyphal cells

In yeast cells of the *chs2*Δ*chs8*Δ mutant, the majority of PI-positive stained cells were dead small buds, indicating that death occurred during the very early stages of polarized bud growth (Table 3). It was reasoned that the class I enzymes should therefore be present at sites of early polarized growth in yeast cells, i.e. at the tips of growing buds. Previously, we had shown that Chs2-YFP and Chs8-YFP localize to the mother-bud neck in yeast cells (Lenardon et al., 2007), but a comprehensive analysis of the dynamics of the localization of these two enzymes had not been undertaken. Therefore, the dynamic localization of Chs2-YFP and Chs8-YFP was examined by live-cell fluorescence microscopy. A combination of single snap-shot images and time-lapse movies of Chs2-YFP (*CHS2-YFP*/*chs2*Δ*0*; Table 1) and Chs8-YFP (*CHS8-YFP* and *CHS8-YFP*/*chs8*Δ*0*; Table 1) in live yeast cells were recorded.

**Fig. 2:**
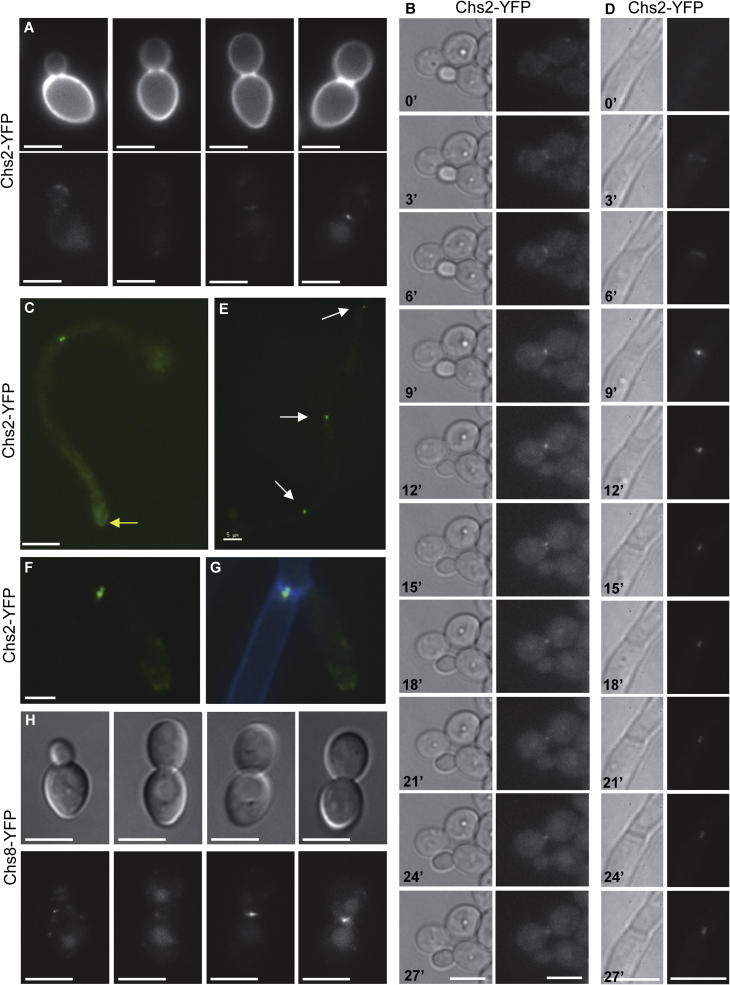
Localization of Chs2-YFP and Chs8-YFP in yeast and hyphal cells. Chs2-YFP was imaged in yeast and hyphae of the *CHS2-YFP*/*chs2*Δ*0* strain (A–G) and Chs8-YFP was imaged in yeast of the *CHS8-YFP* strain (H). A. Single snap-shot images of yeast cells with different sized buds grown in YPD at 30 °C for 4 h and then counterstained with CFW. Chs2-YFP localized to the tip of a small bud (first panel), and as a bar (third panel) and spot (fourth panel) at septa of cells where the mother and bud are approximately equal in size. B. Selected frames of a time-lapse movie of Chs2-YFP in a yeast cell grown on an SC agar pad at 30 °C. Images were recorded every 3 min. C. Single snap-shot images of a hypha grown on an agar pad containing 20% FCS and CFW at 37 °C for 6 h showing the persistent localization of Chs2-YFP at hyphal septa and at hyphal tips (yellow arrow). D. Selected frames of a time-lapse movie of Chs2-YFP in a hypha grown on a 20% FCS agar pad at 37 °C. Images were recorded every 3 min. E. Single snap-shot image of a hypha grown on an agar pad containing 20% FCS and CFW at 37 °C for 6 h showing the persistent localization of Chs2-YFP at hyphal septa (white arrows). F–G. Higher magnification image showing Chs2-YFP localized as two dots (G) on either side of a hyphal septum stained with CFW (G). H. Single snap-shot images of yeast cells with different sized buds grown in YPD at 30 °C for 4 h showing Chs8-YFP localized at septation sites as a bar (third panel) and as a dot (fourth panel) at septa. DIC (B – left panels, D – left panels, H – top panels), YFP (A – bottom panels, B – right panels, C, D – right panels, E, F, H – bottom panels), CFW (A top panels), merge YFP and CFW (G). Scale bars represent 5 μm.

In exponentially growing yeast cells where the bud was approximately one quarter the size of the mother cell, Chs2-YFP signal was observed at the tips of small buds (Fig. 2A first panel), but was not visible in larger buds (Fig. 2A second panel) until cells were about to undergo cytokinesis, i.e. when the mother and bud were approximately equal in size (Fig. 2A third and fourth panels). In these dividing yeast cells, two different patterns of Chs2-YFP localization were visualized at the site of septation; (i) Chs2-YFP was resolved as both a bar (Fig. 2A third panel), and (ii) as a spot in the middle of the septum (Fig. 2A fourth panel). Time-lapse images of Chs2-YFP in yeast cells showed that it localized to septation sites first as a bar (Fig. 2B 3′–6′) which contracted to a spot in the middle of septum (Fig. 2B 9′ onwards). There was no detectable fluorescence signal in the untagged parent control strain (*CHS2*/*chs2*Δ*0*; Table 1) grown and imaged under the same conditions (data not shown).

In hyphae of the *chs2*Δ*chs8*Δ mutant, there were increased proportions of dead short hyphae and dead end compartments compared to wild-type (Table 3). This indicated that the class I enzymes play an important role in early polarized growth, i.e. during initial germ tube formation and re-polarization of growth occurring immediately after hyphal septum formation. Again, we reasoned that the class I enzymes should therefore be present at sites of polarized hyphal growth, i.e. at the tips of growing hyphae. Therefore, the dynamics of the localization of the class I chitin synthases in hyphae was investigated by recording time-lapse movies of Chs2-YFP (*CHS2-YFP*/*chs2*Δ*0*; Table 1) and Chs8-YFP (*CHS8-YFP*/*chs8*Δ*0*; Table 1) in live hyphae (Movies S1–S4).

In hyphae, Chs2-YFP localized to the hyphal tip throughout germ tube growth, including during the early stages of hyphal growth before the first septum was laid down, as can be seen in Movie S1 (corresponding DIC images in Movie S2) and Fig. 2C (arrow). Time-lapse movies of hyphae taken over the duration of septation events (Movie S1 and Fig. 2D) showed that Chs2-YFP again localized to septation sites first as a bar which most likely represents a ring visualized from above (Fig. 2D 3′–6′), which then contracted to a spot in the middle of septum (Fig. 2D 9′) and then separated into two spots, one on each side of the septum (Fig. 2D 12′–27′). Images taken at later time points confirmed that the two trans-septal Chs2-YFP spots persisted in this configuration at hyphal septa throughout subsequent hyphal cell cycles (Fig. 2E). Merged images of Chs2-YFP (Fig. 2F) and CFW fluorescence (Fig. 2G) confirmed that the two Chs2-YFP spots were localized on either side of the chitinous primary septum. Hyphal cells grown on agar without CFW showed the same localization of Chs2-YFP (Movie S1). Therefore the septal localization pattern of Chs2-YFP was similar in yeast and hyphal cells.

Chs8-YFP showed a similar pattern of localization to Chs2-YFP at septa in both yeast and hyphal cells, but fluorescence was of a lower intensity. In exponentially growing yeast cells, Chs8-YFP was only visible at septa when the mother and bud were approximately equal in size, and again localized to septa in two configurations, as a bar (Fig. 2H third panel) and as a spot (Fig. 2H fourth panel). Time-lapse movies of Chs8-YFP in hyphae (Movie S3 (YFP) and Movie S4 (DIC)) revealed a similar pattern of localization at septa as for Chs2-YFP, i.e. Chs8-YFP localized to septa first as a bar which then constricted to a spot that was subsequently maintained in this configuration at septa. Interestingly, Chs8-YFP was not clearly visualized as two spots at septa, and was not observed at hyphal tips.

Therefore, the examination of the dynamic localization of the class I enzymes in live yeast and hyphal cells suggested a link between the localization of Chs2-YFP at the tips of small buds and hyphal tips throughout growth and an important role for the class I enzymes during early polarized growth that was suggested by the PI-positive phenotypes observed in both yeast and hyphal cells. It is likely that the weak intensity of fluorescence of Chs8-YFP has prevented any statement about the presence or absence of Chs8-YFP at hyphal tips, as well as whether it is configured as two spots at septa. The pattern of localization of Chs2-YFP and Chs8-YFP at septa in yeast and hyphal cells suggests that the class I enzymes may also play an important biological function at septation sites.

### 3.3 Phosphorylation of Chs2 on S222 is required for the correct amount of Chs2 at septa and hyphal tips

**Supplementary Fig. S1:**
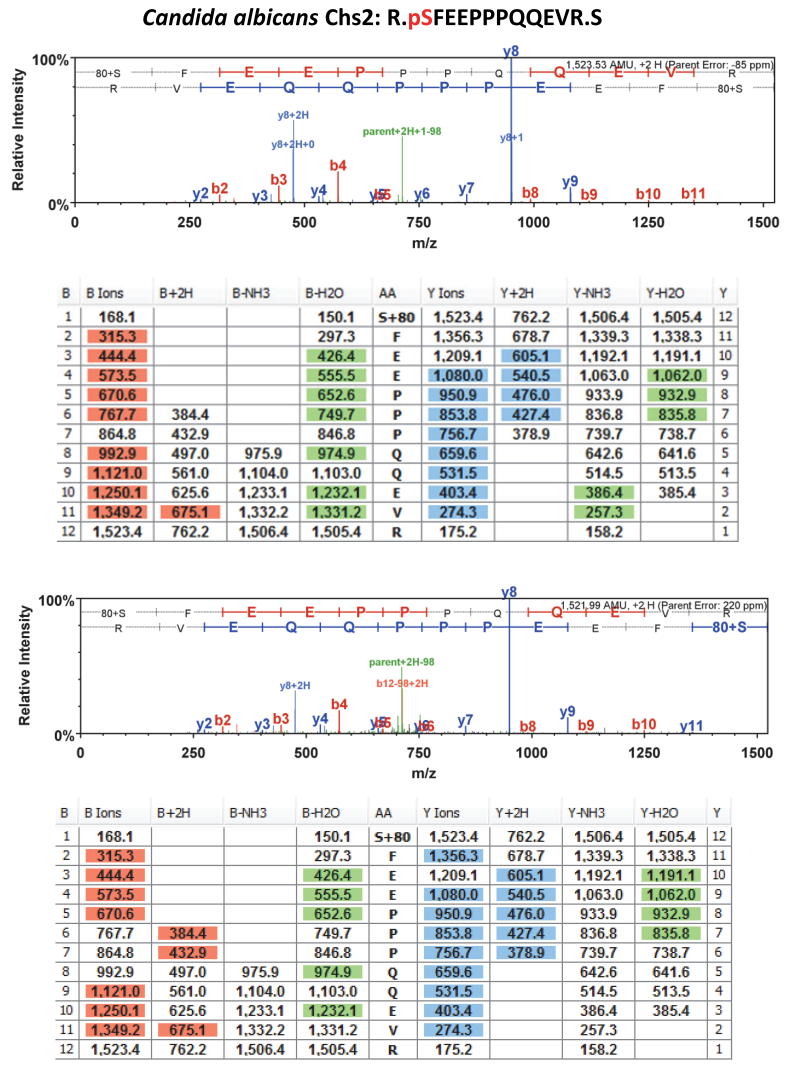
Identification of a phosphorylation site on Chs2. Sequencing of a phosphopeptide from Chs2 by nano-electrospray ionisation tandem mass spectrometry (nano-ESI-MS/MS) showed that the serine residue at the N-terminal end of the peptide is phosphorylated. Phosphopeptide preparation and mass spectrometry were performed as described previously (Lenardon et al., 2010a). Data were exported as SRF files into Scaffold (www.proteomesoftware.com) to produce the annotated spectra. Two independent spectra obtained for the same phosphopeptide are shown. In each example, the parent ion after a neutral loss of phosphoric acid is labelled parent+2H-98 (green). The individual y – (blue) and b – (red) ions are labelled on the spectra.

A global analysis of the *C. albicans* phosphoproteome showed that Chs2 is phosphorylated on S222 (Beltrao et al., 2009). This result was confirmed by our own independent phosphoproteomic analysis (Fig. S1). However, the biological consequence, if any, of this post-translational modification is not known. Because the class I chitin synthase enzymes are responsible for the majority of chitin synthase activity that can be measured *in vitro*, we assessed whether phosphorylation of Chs2 on S222 affected the activity of Chs2.

**Fig. 3:**
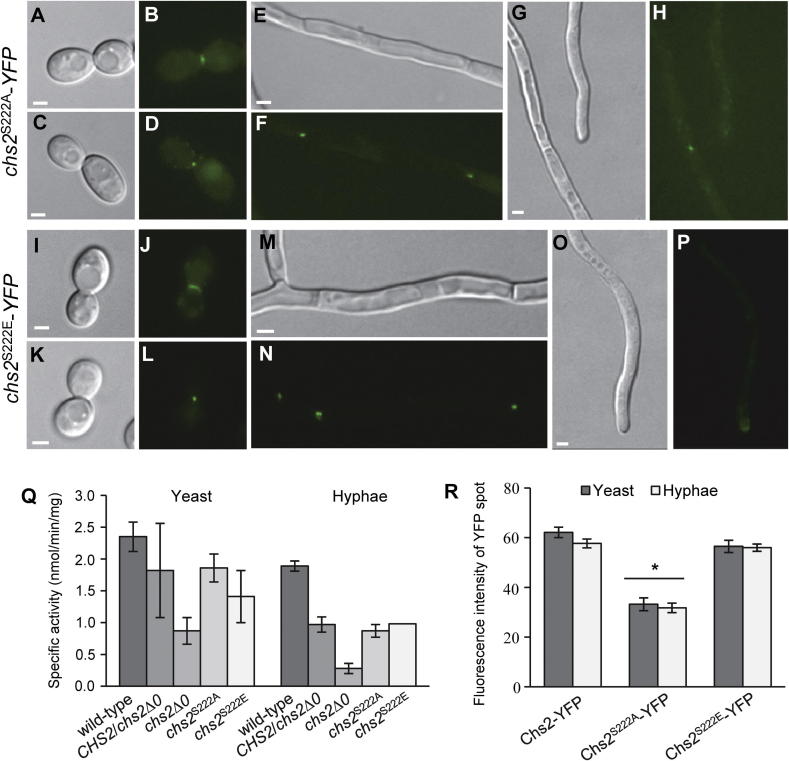
Localization and chitin synthase activity of phosphomutant forms of Chs2. A–P. Yeast cells of the *chs2*^S222A^-*YFP* (A–D) and *chs2*^S222E^-*YFP* strain (I–L) were grown in YPD+uri at 30 °C for 4 h, and hyphae of the *chs2*^S222A^-*YFP* (E–H) and *chs2*^S222E^-*YFP* strain (M–P) were grown on 20% FCS agar pads with uridine at 37 °C for at least 2 h before imaging. Chs2^S222A^-YFP and Chs2^S222E^-YFP was observed at septation sites in yeast cells as a bar (A and B, I and J) and as a spot (C and D, K and L). In hyphae, Chs2^S222A^-YFP and Chs2^S222E^-YFP were observed as two spots at the middle of septa (E–H, M–P). Chs2^S222E^-YFP but not Chs2^S222A^-YFP was observed at hyphal tips (O and P, G and H). Scale bars are 2 μm. Q. Chitin synthase activity of membrane preparations from yeast and hyphal forms of the wild-type (BWP17), *CHS2*/*chs2*Δ*0* heterozygous mutant, *chs2*Δ*0* null mutant, and *chs2*^S222A^ and *chs2*^S222E^ phosphomutant strains. Measurements were made following trypsin treatment of the membrane preparations for two independent biological replicates measured in triplicate (*n* = 6). Error bars are SD. R. The average fluorescence intensity of YFP spots at septa of yeast and hyphae quantified using ImageJ. Error bars are SEM. *n* = 50–80 spots for each measurement. ^∗^ *P* < 0.05 by ANOVA.

Strains that express phospho-mutant versions of Chs2 were constructed by introducing mutations into the remaining *CHS2* allele of the *CHS2*/*chs2*Δ*0* heterozygous mutant (Table 1). A non-phosphorylatable form of Chs2 was created by mutating S222 to alanine (S222A) (*chs2*^S222A^; Table 1) and a phospho-mimetic form of Chs2 was also created by introducing an S222E mutation (*chs2*^S222E^; Table 1). The *in vitro* chitin synthase activity in mixed membrane preparations from yeast and hyphae of the phospho-mutant strains was measured. No difference in the specific activity was observed for the phospho-mutant strains (*chs2*^S222A^ and *chs2*^S222E^) compared to their parent strain (*CHS2/chs2*Δ*0*) (Fig. 3Q). Therefore, *in vitro* chitin synthase activity was not dependent on phosphorylation of Chs2 on S222.

**Fig. 4:**
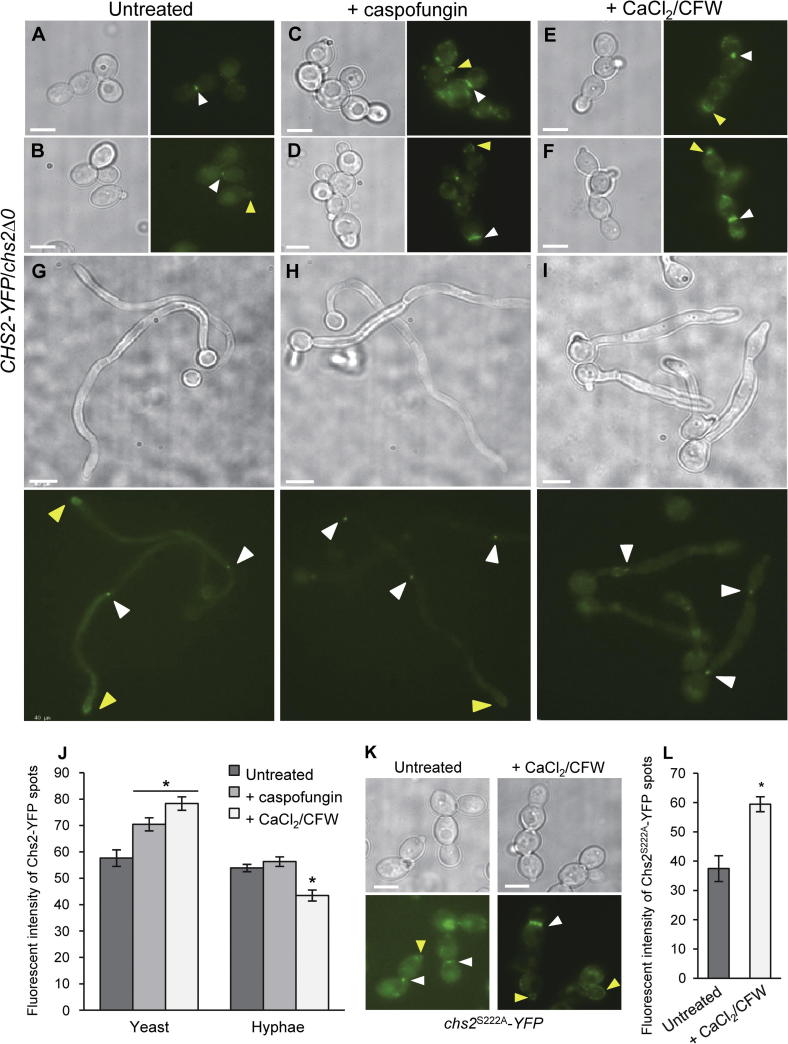
Localization of Chs2-YFP in yeast and hyphal cells grown in the presence of caspofungin and CaCl_2_ and CFW. A–F. Yeast cells of the *CHS2-YFP*/*chs2*Δ*0* strain were grown in YPD (A and B) YPD with a sub-MIC concentration of caspofungin (C and D) or YPD with CaCl_2_/CFW (E and F) at 30 °C for 5 h before imaging. G–I. Hyphae of the *CHS2-YFP*/*chs2*Δ*0* strain were grown on 20% FCS agar pads (G) with a sub-MIC concentration of caspofungin (H) or CaCl_2_/CFW (I) at 37 °C for 4 h before imaging. White arrow heads point to septa, and yellow arrow heads point to the tips of buds and hyphae. Scale bars are 5 μm. J. The average fluorescence intensity of Chs2-YFP spots at septa of yeast and hyphae treated with a sub-MIC concentration of caspofungin or CaCl_2_/CFW quantified using ImageJ. Error bars are SEM. *n* = 40–60 spots for each measurement. ^∗^* p *< 0.05 by ANOVA. K. Yeast cells of the *chs2*^S222A^-*YFP* strain were grown in YPD (Untreated) or YPD with CaCl_2_ and CFW (+CaCl_2_/CFW) at 30 °C for 5 h before imaging. White arrow heads point to Chs2^S222A^-YFP at septa, and yellow arrow heads point to the tips of buds. Scale bars are 5 μm. L. The average fluorescence intensity of Chs2^S222A^-YFP spots at septa of yeast cells treated with a sub-MIC concentration of caspofungin and CaCl_2_/CFW quantified using ImageJ. Error bars are SEM. *n* = 20–40 spots for each measurement. ^∗^ *P* < 0.05 by *t*-test.

It has been shown that phosphorylation of Chs3 on S139 is important for the localization of Chs3-YFP to sites of polarized growth (Lenardon et al., 2010a). We therefore tested whether phosphorylation was involved in regulating the localization of Chs2. To do this, strains expressing C-terminally YFP-tagged phospho-mutant versions of Chs2 (*chs2*^S222A^-YFP and *chs2*^S222E^-YFP; Table 1) were constructed and the localization of Chs2^S222A^-YFP and Chs2^S222E^-YFP was examined by fluorescence microscopy. In single snap-shot images, Chs2^S222A^-YFP was observed at septa of yeast cells as a bar and as a spot (Fig. 3A–D) and at the septa of hyphal cells (Fig. 3E–H) in the same configurations as Chs2-YFP (Fig. 2A–G). However, Chs2^S222A^-YFP was not observed at hyphal tips (Fig. 3G and H). Chs2^S222E^-YFP was also observed in the same configuration as Chs2-YFP at the septa of yeast (Fig. 3I–L) and hyphal cells (Fig. 4M and N), as well as at hyphal tips (Fig. 3O and P). These results indicate that either phosphorylation on S222 is required for Chs2 to be localized to hyphal tips, or that the amount of Chs2^S222A^-YFP at hyphal tips was reduced to a non-detectable level in this mutant.

An estimate of the amount of the YFP-tagged versions of Chs2 at septa was made by measuring the intensity of YFP fluorescence when the protein was visible as a single spot at the septum using ImageJ. This analysis suggested that the intensity of Chs2^S222A^-YFP spots was approximately 50% less than that of Chs2-YFP spots at both yeast and hyphal septa (Fig. 3R). Therefore, phosphorylation of Chs2 on S222 appears to be required for the normal localization of this chitin synthase at septa, and phosphorylation of Chs2 on S222 regulates the amount of Chs2 that is directed to specific sites in both yeast and hyphal cells.

### 3.4 Cell wall stresses increase the targeting of Chs2 to septa and budding tips in yeast cells

Transcription of the genes encoding the class I chitin synthases (*CHS2* and *CHS8*) are the most highly up-regulated of the chitin synthase genes in *C. albicans* yeast cells treated with caspofungin or CaCl_2_ and CFW (CaCl_2_/CFW). This transcriptional up-regulation correlates with an increase in the *in vitro* chitin synthase activity (Munro et al., 2007; Walker et al., 2008). Therefore, another proposed role for the class I enzymes is in providing protection to stressed cells by reinforcing the cell wall with chitin. We therefore tested whether this resulted in changes in the localization of these enzymes in cells treated with caspofungin or CaCl_2_/CFW.

Cells of the *CHS2-YFP*/*chs2*Δ*0* strain (Table 1) were grown in YPD containing a sub-MIC concentration of caspofungin or in CaCl_2_/CFW. Chs2-YFP was visualized by fluorescence microscopy. In yeast cells, Chs2-YFP was observed in punctate patches all over the membrane of budding tips in the presence of caspofungin or CaCl_2_/CFW (Fig. 4C–F, yellow arrow heads), and the YFP fluorescence at septa was brighter (Fig. 4C–F, white arrow heads) than in cells grown in the absence of caspofungin or CaCl_2_/CFW (Fig. 4A and B, Untreated). The average intensity of fluorescence of Chs2-YFP spots at septa in cells was measured and we found that the YFP-fluorescence was, on average, 22% and 36% higher in cells treated with caspofungin or CaCl_2_/CFW than in untreated cells (Fig. 4J, yeast). This indicates that there was increased targeting of Chs2-YFP to septa and budding tips in yeast cells grown in the presence of caspofungin or CaCl_2_/CFW.

Since treatment with CaCl_2_/CFW and phosphorylation of Chs2 on S222 both affected the amount of Chs2-YFP observed at septa in yeast cells, we assessed whether there was a functional relationship between these two events. We reasoned that if the mechanism for localizing more Chs2-YFP to septa in the presence of CaCl_2_/CFW was dependent on the phosphorylation of Chs2 on S222, then there may not be an increase in the amount of Chs2^S222A^-YFP at septa in CaCl_2_/CFW-treated cells compared to untreated cells because Chs2^S222A^ cannot be phosphorylated. However, if the mechanism for localizing more Chs2-YFP to septa in the presence of CaCl_2_/CFW was not dependent on the phosphorylation of Chs2 on S222, then the amount of Chs2^S222A^-YFP at septa should increase as normal after treatment.

To assess whether there was an increase in the amount of Chs2^S222A^-YFP at septa in cells treated with CaCl_2_/CFW, cells of the *chs2*^S222A^*-YFP* strain (Table 1) were grown in YPD containing CaCl_2_/CFW and Chs2^S222A^-YFP was visualized by fluorescence microscopy. Chs2^S222A^-YFP was observed in punctate patches at the periphery of growing buds (yellow arrow heads) in yeast cells that were treated with CaCl_2_/CFW but not in untreated cells (Fig. 4K). The average intensity of fluorescence of Chs2^S222A^-YFP spots at septa was 59% higher in cells treated with CaCl_2_/CFW than untreated cells (Fig. 4L). Taken together, these results show that treatment with CaCl_2_/CFW resulted in increased targeting of Chs2-YFP to the tips of buds and septa in yeast cells, but this was not dependent on the phosphorylation of Chs2 on S222.

To assess whether there was also a change in the localization of Chs2-YFP in hyphal cells treated with caspofungin or CaCl_2_/CFW, hyphae of the *CHS2-YFP*/*chs2*Δ*0* strain (Table 1) were grown in 20% FCS containing a sub-MIC concentration of caspofungin or CaCl_2_/CFW, and Chs2-YFP was visualized by fluorescence microscopy. In hyphae, Chs2-YFP was observed at septa (Fig. 4G and H, white arrow heads) and tips (Fig. 4G and H, yellow arrow heads) when grown in the absence or presence of a sub-MIC dose of caspofungin. However, measurement of the average fluorescence intensity of Chs2-YFP spots at septa indicated that there was no increase in the amount of Chs2-YFP at septa of cells grown in the presence of caspofungin compared to untreated cells (Fig. 4J). The presence of CaCl_2_/CFW in the growth medium influenced cellular morphology and cells grew as pseudohyphae (Fig. 4I). Chs2-YFP was observed at septation sites (Fig. 4I, white arrow heads) but not at tips. However, the average fluorescence intensity of Chs2-YFP spots at septa in cells grown in the presence of CaCl_2_/CFW was 20% lower than in untreated cells (Fig. 4J, hyphae). Therefore the localization of Chs2-YFP in hyphae was not affected by treatment with a sub-MIC concentration of caspofungin, and treatment with CaCl_2_/CFW affected hyphal morphogenesis and resulted in a reduction in the amount of Chs2-YFP at septa.

### 3.5 The class I chitin synthases provide protection to yeast and hyphal cells during exposure to cell wall stresses

To understand the function of the class I chitin synthases under cell wall stress conditions in more detail, we next assessed whether the class I chitin synthases provided protection to yeast cells grown in the presence of CaCl_2_/CFW or caspofungin using PI staining. Yeast cells of the wild-type (CAI-4; Table 1) and *chs2*Δ*chs8*Δ mutant strains (Table 1) were grown in YPD+uri containing CaCl_2_/CFW or a sub-MIC of caspofungin for 5 h at 30 °C, counter-stained with PI, and imaged by fluorescence microscopy. These culture conditions matched those used to assess the changes in the localization of Chs2-YFP in the presence of these cell wall stresses. The PI-positive phenotypes that were observed in the previous analysis of wild-type and *chs2*Δ*chs8*Δ mutant yeast cells (Fig. 1A–D and Table 2) were also observed in these experiments (Table 4).

**Fig. 5:**
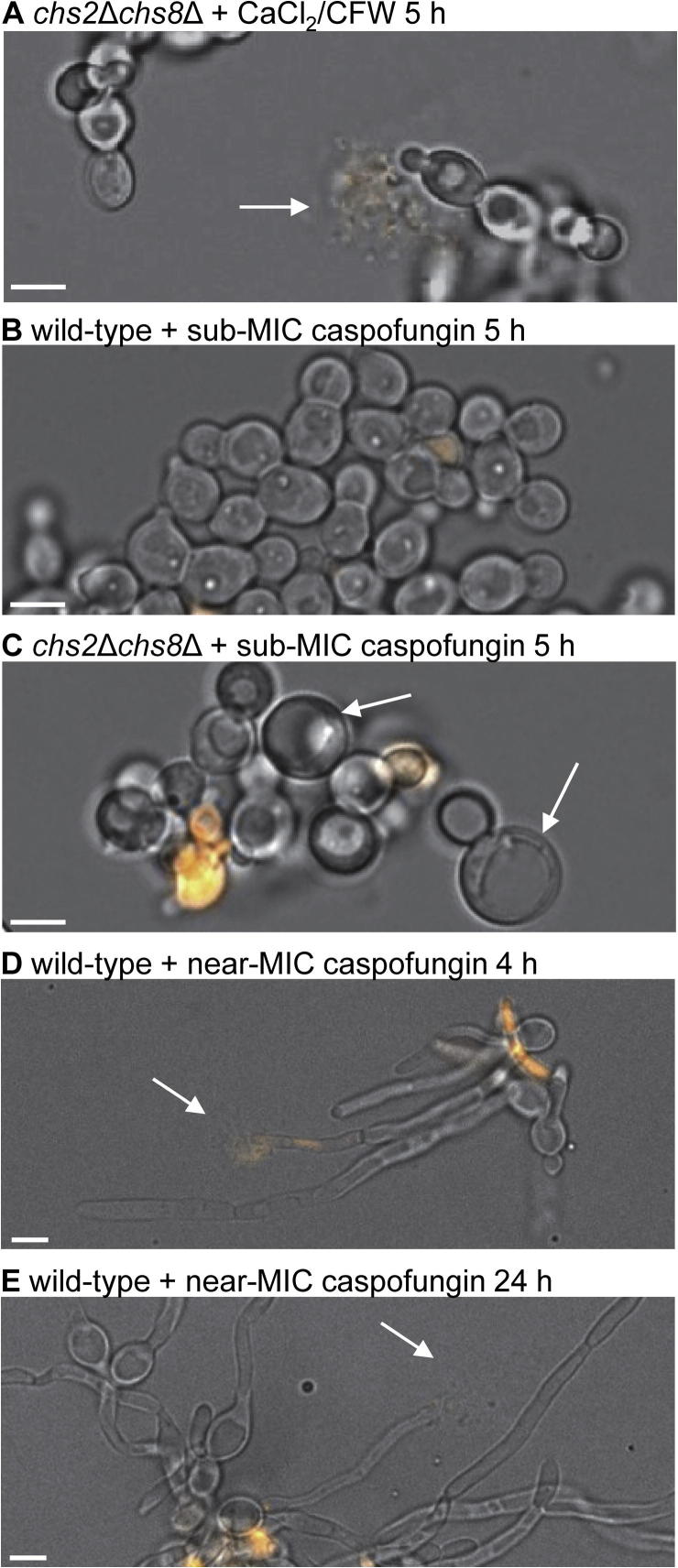
Additional phenotypes observed in yeast and hyphae grown in the presence of CaCl_2_ and CFW or caspofungin. A-C. Yeast cells of the *chs2*Δ*chs8*Δ mutant and wild-type (CAI-4) strains were grown in YPD+uri + CaCl_2_/CFW (A) or YPD + sub-MIC caspofungin (B and C) for 5 h at 30 °C and counterstained with PI. Panels show representative images of lysis events (A, arrow), irregular shaped cells (B) and giant cells (C, arrows) observed in a proportion of cells grown under these conditions. D and E. A proportion of hyphae of the wild-type strain (CAI-4) grown on 20% FCS agar pads with PI and a near-MIC concentration of caspofungin at 37 °C for either 4 h (C) or 24 h (D) displayed a hyphal tip-lysis phenotype (arrows). All panels show the overlay of PI fluorescence on the corresponding DIC image. Scale bars represent 5 μm.

After 5 h growth in the absence of any cell wall stress, less than 1% of cells wild-type and *chs2*Δ*chs8*Δ mutant strains stained PI-positive with no difference between the wild-type and mutant strains (Table 4). When grown in the presence of CaCl_2_/CFW for 5 h, a slight increase in the over-all percentage of cells that stained PI-positive in both the wild-type and *chs2*Δ*chs8*Δ mutant strains compared to untreated cells was observed (1.5–2% for treated compared to <1% for untreated cells; Table 4), although there was no difference in the overall percentage of PI-positive stained cells between the wild-type and *chs2*Δ*chs8*Δ mutant strains (Table 4). However, evidence of cell lysis was observed in the *chs2*Δ*chs8*Δ mutant grown in the presence of CaCl_2_/CFW (Fig. 5A) that was never observed in wild-type cells. These lysis events were observed on 16 separate occasions representing 0.79 ± 0.14% of *chs2*Δ*chs8*Δ mutant cells counted in two independent biological replicates (*n* = 1344 and 577). This indicates that the class I enzymes protect yeast cells from lysis when grown in the presence of CaCl_2_/CFW.

An increase in the percentage of dead small buds of the *chs2*Δ*chs8*Δ mutant compared to the wild-type was observed when grown in the presence of a sub-MIC of caspofungin for 5 h (Table 4). Differences in the morphology of the cells were also observed in the presence of caspofungin. Wild-type cells tended to be an irregular shape with a wide mother-bud neck (Fig. 5B), where as cells of the *chs2*Δ*chs8*Δ mutant were unusually round, and giant cells were observed (Fig. 5C). The giant cells had a diameter that was 1.5–2 × larger than normal cells, and comprised 13.51 ± 2.54% of *chs2*Δ*chs8*Δ mutant cells that were counted in two independent biological replicates (*n* = 601 and 572). These results indicate that Chs2 and Chs8 provide protection to yeast cells during the early stages of bud growth in the presence of caspofungin, and affect cellular morphology under this cell wall stress condition.

We also used PI staining to investigate whether the class I chitin synthases provide protection to hyphal cells grown in the presence of caspofungin. Hyphal growth of the wild-type (CAI-4; Table 1) and *chs2*Δ*chs8*Δ mutant strain (Table 1) was induced on agar pads containing 20% FCS, PI and a sub-MIC or near-MIC concentration of caspofungin at 37 °C for 4 h and 24 h and cells were imaged by fluorescence microscopy.

The PI-positive phenotypes that were observed in the previous analysis of wild-type and *chs2*Δ*chs8*Δ mutant hyphae (Fig. 1E–I and Table 3) were also observed when these cells were grown in the presence of a sub-MIC concentration of caspofungin. However, no difference in the distribution of the frequencies of these PI-positive phenotypes was observed when *chs2*Δ*chs8*Δ mutant hyphae were grown in the presence of a sub-MIC dose of caspofungin compared to wild-type cells grown in the presence of the same concentration of caspofungin (data not shown).

At near-MIC concentrations of caspofungin, an additional PI-positive phenotype was observed, i.e. hyphal tip lysis (Lysed tips, Table 5 and Fig. 5D,E). Over all, the distribution of the frequencies of these PI-positive phenotypes that were observed in wild-type and *chs2*Δ*chs8*Δ mutant hyphae grown in the presence of caspofungin were significantly different after both 4 h (Chi-squared = 119.17, 7 degrees of freedom, *P* < 0.005) and 24 h (Chi-squared = 302.18, 7 degrees of freedom, *P* < 0.005). Further statistical analyses revealed other significant differences. After 4 h, there were 2.5 × more dead short hyphae, 5.75 × less lysed tips and 1.6 × less live hyphae in the *chs2*Δ*chs8*Δ mutant compared to wild-type grown in the presence of caspofungin (Table 4). After 24 h, caspofungin treatment resulted in 5.4 × more dead short hyphae and 8.6 × less lysed tips in the *chs2*Δ*chs8*Δ mutant compared to wild-type (Table 5). These results therefore show that a near-MIC concentration of caspofungin killed more *chs2*Δ*chs8*Δ mutant hyphae in earlier stages of growth than wild-type hyphae. The overall decrease in the proportion of live hyphae at 4 h and 24 h reinforces the hypothesis that the class I enzymes provide protection to hyphal cells during exposure to cell wall stresses.

## 4 Discussion

Determining the biological function of class I enzymes based on the phenotypes of mutants in fungi has proved difficult. Some filamentous fungi have many class I enzymes, and their functional analysis has been confounded by the potential for functional redundancy and/or overlapping functions. *C. albicans* has two class I enzymes and the significance of neither of these has been fully established.

In *Aspergillus nidulans*, deletion of the gene encoding the class I chitin synthase (*An*ChsC) did not result in any obvious phenotypes (Motoyama et al., 1994). In *Wangiella dermatitidis* and *Saccharomyces cerevisiae*, deletion of the genes encoding the class I enzymes (*Wd*Chs2 and *Sc*Chs1) resulted in an 85% and a 90% reduction in the *in vitro* chitin synthase activity (Bulawa et al., 1986; Wang et al., 2001). In *S. cerevisiae*, the class I chitin synthase *Sc*Chs1 plays an important role in protecting the integrity of yeast cells at cytokinesis (Bulawa et al., 1986; Cabib and Arroyo, 2013; Cabib et al., 1989). Chitinase-dependent lysis of a proportion of nascent buds was observed in an *Scchs1* mutant, suggesting that *Sc*Chs1 acts as a repair enzyme, replenishing chitin in the septum as the mother and bud separate due to the local action of cell wall hydrolases at the division site (Cabib et al., 1989).

In *C. albicans*, cell lysis was not observed in *chs2*Δ or *chs2*Δ*chs8*Δ mutant yeast cells in previous studies which used cell refractivity or Trypan Blue exclusion as methods to assess cell viability (Gow et al., 1994; Munro et al., 2003). In this work, the fluorescent dye PI was used because it increases the sensitivity of cell viability assays to explore chitin synthase function (Ali et al., 2010; Jones and Senft, 1985; Kaloriti et al., 2012; Mascotti et al., 2000; Ramani et al., 1997). This allowed us to observe clear phenotypes for the class I mutants and make inferences relating to the biological functions for these enzymes during normal growth conditions and under conditions of cell wall stress.

Our previous studies on Chs3 in *C. albicans* showed that the phenotype of a *chs3*Δ mutant was reflected in its cellular localization (Lenardon et al., 2010a, 2007). In this work we again show a strong correlation between the localization of Chs2-YFP at sites of polarized growth, i.e. the tips of small buds and hyphae, and the most prevalent PI-positive phenotypes, i.e. small dead buds and dead short hyphae.

We observed a unique pattern of localization of the class I enzymes at septation sites. This revealed for the first time a persistent localization of a chitin synthase at septation sites. However, no PI-positive phenotypes that correlate specifically with a defect in septum formation were observed. A striking difference between wild-type and *chs2*Δ*chs8*Δ mutant yeast cells was the presence of dead small buds in the class I mutant which were never observed in wild-type cells (Table 2). In this case, death of the bud occurred well before septum formation had initiated, an event which, at least in *S. cerevisiae*, is triggered by mitotic exit and does not occur until the mother and bud are approximately equal in size (reviewed in Wloka and Bi (2012)). Similarly in hyphae observed after 4 h of growth, i.e. a similar time frame to that in which the movies were recorded, apical compartments which stained PI-positive were observed *chs2*Δ*chs8*Δ mutant hyphae (Fig.1H) but never in wild-type hyphae (Table 3). In these hyphae, the PI staining did not extend to the adjacent compartment, indicating the presence of a complete and sealed septal barrier.

As yet, there is no direct evidence to link the localization of the class I enzymes at septa with a specific function at these sites under normal growth conditions. However, because Chs8 is required for the synthesis of long chitin microfibrils observed in the bud scars of yeast cells and septal plates of hyphae (Lenardon et al., 2007), it has been hypothesized that this enzyme may co-operate with Chs1 during the synthesis of the primary chitinous septum and affect the overall architecture of chitin microfibrils that are formed in the primary septum. Additionally, since pores have been previously observed in hyphal septa (Gooday and Gow, 1983; Gow et al., 1980), the class I enzymes, which persist at hyphal septa, might be involved in the closure of septal pores. If Chs2 and Chs8 were required for the closure of septal pores, then these pores may be expected to remain open in the septa of hyphae of the *chs2*Δ*chs8*Δ mutant. However, after 24 h of growth, we did not observe adjacent PI-positively stained hyphal compartments along the entire length of a *chs2*Δ*chs8*Δ mutant hypha, suggesting that septa were sealed. It is possible that defects in the sealing of septa would not be observed because other chitin synthase (Chs1 or Chs3) could seal septal pores in the absence of the class I enzymes (e.g. see Walker et al. (2013)). Further investigations are required to elucidate the precise role of the class I enzymes at septation sites.

By constructing strains that express phospho-mutant versions of Chs2, we determined that phosphorylation of Chs2 on S222 does not influence the measured *in vitro* activity of the enzyme, but instead, may affect amount of protein that is localized to specific sites of cells. Quantification of the intensity of fluorescence of YFP-tagged versions of wild-type and phospho-mutant forms of Chs2 at septa, we provided evidence that phosphorylation of Chs2 on S222 may regulate the amount of Chs2-YFP that is localized to septation sites. Further investigations are required to identify the kinase(s) that phosphorylate Chs2 on S222.

This work has also provided evidence that increased targeting of the class I enzymes to budding tips and septa provides protection to yeast cells grown in the presence of a sub-MIC concentration of caspofungin or CaCl_2_/CFW. More specifically, the class I enzymes protect yeast cells from death during the early stages of budding and prevent the formation of giant cells when grown in the presence of caspofungin, and protect yeast cells from lysis when grown in the presence of CaCl_2_/CFW. The concentration of CaCl_2_/CFW used in our experiments was chosen to be consistent with previous studies that linked transcriptional activation of the class I genes with increased *in vitro* chitin synthase activity in yeast cells (Munro et al., 2007). We also showed that the mechanism for targeting Chs2-YFP to the tips of growing buds and septa in the presence of CaCl_2_/CFW was not directly related to phosphorylation of Chs2 on S222.

In hyphae, we were unable to image Chs2-YFP in hyphae grown in a near-MIC concentration of caspofungin. However, we demonstrated an indirect relationship between the class I enzymes and protection from caspofungin, particularly during the early stages of hyphal growth. This was evident in the analysis of PI-positive phenotypes of wild-type and *chs2*Δ*chs8*Δ mutant hyphae grown in the presence of a near-MIC concentration of caspofungin. More specifically, in the presence of caspofungin, the absence of the class I enzymes resulted in a statistically significant increase in the proportion of hyphae that died before the first septum was laid down (i.e. dead short hyphae), and a decrease in the proportion of hyphae that died after laying down two or three septa (i.e. lysed tips). The observed decrease in the proportion of cells that died in later stages of hyphal growth may simply be due to the higher proportion of hyphae that died during the early stages of hyphal growth. This analysis reinforced the importance of the role of class I enzymes during the early stages of polarized growth.

In summary, we have demonstrated a new role for the *C. albicans* class I chitin synthases in promoting and maintaining cell integrity during early polarized growth in yeast and hyphal cells, both under normal growth conditions, and during conditions of cell wall stress. We also describe a unique localization pattern for these enzymes at septa in live yeast and hyphal cells, and demonstrate that phosphorylation of Chs2 on S222 and specific cell wall stresses independently regulate the amount of Chs2 that is localized to specific sites in cells. These observations demonstrate that the *C. albicans* class I chitin synthases contribute to the stabilization and protection of the cell wall during normal growth and during conditions of cell wall stress.

## Figures and Tables

**Table 1:** *C. albicans* strains used and constructed in this study.

Strain name	Published name	Genotype	Reference
CAI-4	CAI-4	*ura3*Δ::*λimm434*/*ura3*Δ::*λimm434*	Fonzi and Irwin (1993)
*chs2*Δ	C155	*ura3*Δ::*imm434*/*ura3*Δ::*imm434, chs2*Δ::*hisG*/*chs2*Δ::*hisG*	Mio et al. (1996)
*chs8*Δ	NGY128	*ura3*Δ::*imm434*/*ura3*Δ::*imm434, chs8*Δ::*hisG*/*chs8*Δ::*hisG*	Munro et al. (2003)
*chs2*Δ*chs8*Δ	NGY138	*ura3*Δ::*imm434*/*ura3*Δ::*imm434, chs2*Δ::*hisG*/*chs2*Δ::*hisG, chs8*Δ::*hisG*/*chs8*Δ::*hisG*	Munro et al. (2003)
BWP17	BWP17	*ura3*Δ::*imm434*/*ura3*Δ::*imm434, his1*Δ::*hisG*/*his1*Δ::*hisG, arg4*Δ::*hisG*/*arg4*Δ::*hisG*	Wilson et al. (1999)
*CHS2*/*chs2*Δ*0*	NGY479	*ura3*Δ::*imm434*/*ura3*Δ::*imm434, his1*Δ::*hisG*/*his1*Δ::*hisG, arg4*Δ::*hisG*/*arg4*Δ::*hisG, CHS2*/*chs2*Δ*0*::*CdHIS1*	Lenardon et al. (2007)
*chs2*Δ*0*	NGY603	*ura3*Δ::*imm434*/*ura3*Δ::*imm434, his1*Δ::*hisG*/*his1*Δ::*hisG, arg4*Δ::*hisG*/*arg4*Δ::*hisG, chs2*Δ*0*::*CdHIS1*/*chs2*Δ*0*::*dpl200*	This study
*chs2*^S222A^	NGY604	*ura3*Δ::*imm434*/*ura3*Δ::*imm434, his1*Δ::*hisG*/*his1*Δ::*hisG, arg4*Δ::*hisG*/*arg4*Δ::*hisG, chs2*Δ*0*::*CdHIS1*/*chs2*^S222A^	This study
*chs2*^S222E^	NGY605	*ura3*Δ::*imm434*/*ura3*Δ::*imm434, his1*Δ::*hisG*/*his1*Δ::*hisG, arg4*Δ::*hisG*/*arg4*Δ::*hisG, chs2*Δ*0*::*CdHIS1*/*chs2*^S222E^	This study
*CHS2-YFP*/*chs2*Δ*0*	NGY481	*ura3*Δ::*imm434*/*ura3*Δ::*imm434, his1*Δ::*hisG*/*his1*Δ::*hisG, arg4*Δ::*hisG*/*arg4*Δ::*hisG, CHS2-YFP-URA3*/*chs2*Δ*0*::*CdHIS1*	Lenardon et al. (2007)
*chs2*^S222A^*-YFP*	NGY606	*ura3*Δ::*imm434*/*ura3*Δ::*imm434, his1*Δ::*hisG*/*his1*Δ::*hisG, arg4*Δ::*hisG*/*arg4*Δ::*hisG, chs2*Δ*0*::*CdHIS1*/*chs2*^S222A^*-YFP-URA3*	This study
*chs2*^S222E^*-YFP*	NGY607	*ura3*Δ::*imm434*/*ura3*Δ::*imm434, his1*Δ::*hisG*/*his1*Δ::*hisG, arg4*Δ::*hisG*/*arg4*Δ::*hisG, chs2*Δ*0*::*CdHIS1*/*chs2*^S222E^*-YFP-URA3*	This study
*CHS8-YFP*	NGY478	*ura3*Δ::*imm434*/*ura3*Δ::*imm434, his1*Δ::*hisG*/*his1*Δ::*hisG, arg4*Δ::*hisG*/*arg4*Δ::*hisG, CHS8-YFP-URA3*/*CHS8, RPS1*/*RPS1*::CIp30	Lenardon et al. (2007)
*CHS8*/*chs8*Δ*0*	NGY609	*ura3*Δ::*imm434*/*ura3*Δ::*imm434, his1*Δ::*hisG*/*his1*Δ::*hisG, arg4*Δ::*hisG*/*arg4*Δ::*hisG, CHS8*/*chs8*Δ*0*::*CdHIS1*	This study
*chs8*Δ*0*	NGY608	*ura3*Δ::*imm434*/*ura3*Δ::*imm434, his1*Δ::*hisG*/*his1*Δ::*hisG, arg4*Δ::*hisG*/*arg4*Δ::*hisG, chs8*Δ*0*::*CdHIS1*/*chs8*Δ*0*::*dpl200*	This study
*CHS8-YFP*/*chs8*Δ*0*	NGY602	*ura3*Δ::*imm434*/*ura3*Δ::*imm434, his1*Δ::*hisG*/*his1*Δ::*hisG, arg4*Δ::*hisG*/*arg4*Δ::*hisG, CHS8-YFP-URA3*/*chs8*Δ*0*::*CdHIS1*	This study

**Table 2:** Percentage of PI-positive stained yeast cells after growth in SD broth for 24 h at 30 °C.

Strain	Small bud^a^	Large bud^b^	Both mother-and-bud and unbudded cells^c^	Total ± SD
wild-type (CAI-4)	0.00	2.33	1.38	3.71 ± 1.9
*chs2*Δ	2.88	0.92	1.83	5.62 ± 0.9
*chs8*Δ	0.38	0.14	1.83	2.35 ± 0.4
*chs2*Δ*chs8*Δ	3.94	2.99	2.35	9.28 ± 5.2

The percentage is the average of two independent biological replicates (*n* = 250–750 per replicate). Errors are SD.

a Where the bud is less than one fourth of the size of the mother cell (Fig. 1A and B).

b Where the bud is greater than one fourth of the size of the mother cell (Fig.1C).

c Where the bud and mother are PI-positive stained cells (counted as two cells) (Fig. 1D).

**Table 3:** Distribution of PI-positive phenotypes in hyphae after 4 h and 24 h incubation at 37 °C.

Phenotype	Time	Strain			
wild-type (CAI-4)		*chs2*Δ*chs8*Δ	
Counts	%	Counts	%
Dead mothers^a^	4 h	9	1.77	25	3.77
Dead buds^b^		20	3.94	35	5.27
Dead short hyphae^c^		1	0.20	25	3.77^∗^
Dead end compartments^d^		0	0.00	14	2.11^∗^
Dead mid-compartments^e^		0	0.00	1	0.15
Live yeast		2	0.39	11	1.66
Live hyphae		476	93.70	553	83.28

Total cells		508	100.00	664	100.00
					
Dead mothers^a^	24 h	20	3.83	7	1.18
Dead buds^b^		9	1.72	12	2.02
Dead short hyphae^c^		4	0.77	17	2.86^∗^
Dead end compartments^d^		9	1.72	6	1.01
Dead mid-compartments^e^		1	0.19	0	0.00
Live yeast		0	0.00	0	0.00
Live hyphae		479	91.76	552	92.93

Total cells		522	100.00	594	100.00

^a^Fig. 1E, ^b^Fig. 1F, ^c^Fig. 1G, ^d^Fig. 1H, ^e^Fig. 1I. Counts are the total number of hyphae observed displaying a particular phenotype in three independent biological replicates. % is the count expressed as a percentage of the total number of cells counted. ^∗^ Indicates a statistically significant difference in the proportion of cells displaying a phenotype between the two strains (*P* ⩽ 0.05 by Mann–Whitney *U* test).

**Table 4:** Percentage of PI-positive stained yeast cells after growth in YPD+uri ± CaCl_2_/CFW or YPD+uri ± sub-MIC caspofungin for 5 h at 30 °C.

Strain	Small bud^a^	Large bud^b^	Both mother-and-bud and unbudded cells^c^	Total ± SD
wild-type (CAI-4)	0.15	0.15	0.00	0.29 ± 0.41
*chs2*Δ*chs8*Δ	0.23	0.00	0.69	0.92 ± 0.44
wild-type (CAI-4) + CaCl_2_/CFW	0.88	0.06	1.00	1.94 ± 0.85
*chs2*Δ*chs8*Δ + CaCl_2_/CFW	0.37	0.04	1.02	1.42 ± 0.44
wild-type (CAI-4) + CAS	0.94	2.19	9.48	12.61 ± 4.73
*chs2*Δ*chs8*Δ + CAS	3.80	1.45	10.45	15.70 ± 0.79

The percentage is the average of two independent biological replicates (*n* = 320–1350 per replicate). Errors are SD.

a Where the bud is less than one fourth of the size of the mother cell (Fig. 1A and B).

b Where the bud is greater than one fourth of the size of the mother cell (Fig. 1C).

c Where the bud and mother are PI-positive stained cells (Fig. 1D).

**Table 5:** Distribution of PI-positive phenotypes in hyphae after 4 h and 24 h incubation at 37 °C in the presence of a near-MIC concentration of caspofungin.

Phenotype	Time	Strain			
wild-type (CAI-4) + CAS		*chs2*Δ*chs8*Δ + CAS	
Counts	%	Counts	%
Dead mothers^a^	4 h	48	11.40	28	5.13
Dead buds^b^		23	5.46	25	4.58
Dead short hyphae^c^		92	21.85	297	54.40^∗^
Dead end compartments^d^		13	3.09	14	2.56
Dead mid-compartments^e^		0	0.00	0	0.00
Lysed tips^f^		31	7.36	7	1.28^∗^
Live yeast		10	2.38	12	2.20
Live hyphae		204	48.46	163	29.85^∗^

Total		421	100.00	546	100.00
					
Dead mothers^a^	24 h	60	7.98	31	6.07
Dead buds^b^		22	2.93	17	3.33
Dead short hyphae^c^		66	8.78	241	47.16^∗^
Dead end compartments^d^		20	2.66	38	7.44
Dead mid-compartments^e^		36	4.79	5	0.98
Lysed tips^f^		63	8.38	5	0.98^∗^
Live yeast		5	0.66	5	0.98
Live hyphae		480	63.83	169	33.07

Total		752	100.00	511	100.00

^a^Fig. 1E, ^b^Fig. 1F, ^c^Fig. 1G, ^d^Fig. 1H, ^e^Fig. 1I, ^f^Fig. 5D and E. Counts are the total number of hyphae observed displaying a particular phenotype in three independent biological replicates. % is the count expressed as a percentage of the total number of cells counted. ^∗^ Indicates a statistically significant difference in the proportion of cells displaying a phenotype between the two strains grown in the presence of caspofungin (*P* ⩽ 0.05 by Mann–Whitney *U* test).

## Appendix A Supplementary material

Supplementary Table S1 Oligonucleotide primers used in this study.

Supplementary Movie S1 Time-lapse movie showing the localization of Chs2-YFP in a growing hypha of the *CHS2-YFP*/*chs2*Δ*0* strain. Images were recorded every 3 min for 93 min Scale bar represents 5 μm.

Supplementary Movie S2 Corresponding DIC channel of Movie S1.

Supplementary Movie S3 Time-lapse movie showing the localization of Chs8-YFP in a growing hypha of the *CHS8-YFP*/*chs8*Δ*0* strain. Images were recorded every 2 min for 30 min. Scale bars represent 20 μm.

Supplementary Movie S4 Corresponding DIC channel of Movie S3.

## References

[b0005] Ali I., Khan F.G., Suri K.A., Gupta B.D., Satti N.K., Dutt P., Afrin F., Qazi G.N., Khan I.A. (2010). *In vitro* antifungal activity of hydroxychavicol isolated from *Piper betle* L. Ann. Clin. Microbiol. Antimicrob..

[b0010] Beltrao P., Trinidad J.C., Fiedler D., Roguev A., Lim W.A., Shokat K.M., Burlingame A.L., Krogan N.J. (2009). Evolution of phosphoregulation: comparison of phosphorylation patterns across yeast species. PLoS Biol..

[b0015] Bulawa C.E., Miller D.W., Henry L.K., Becker J.M. (1995). Attenuated virulence of chitin-deficient mutants of *Candida albicans*. Proc. Natl. Acad. Sci. USA.

[b0020] Bulawa C.E., Slater M., Cabib E., Au-Young J., Sburlati A., Adair W.L., Robbins P.W. (1986). The *S. cerevisiae* structural gene for chitin synthase is not required for chitin synthesis *in vivo*. Cell.

[b0025] Cabib E., Arroyo J. (2013). How carbohydrates sculpt cells: chemical control of morphogenesis in the yeast cell wall. Nat. Rev. Microbiol..

[b0030] Cabib E., Sburlati A., Bowers B., Silverman S.J. (1989). Chitin synthase 1, an auxiliary enzyme for chitin synthesis in *Saccharomyces cerevisiae*. J. Cell Biol..

[b0035] Fonzi W.A., Irwin M.Y. (1993). Isogenic strain construction and gene mapping in *Candida albicans*. Genetics.

[b0040] Gerami-Nejad M., Berman J., Gale C.A. (2001). Cassettes for the PCR-mediated construction of green, yellow, and cyan fluorescent protein fusions in *Candida albicans*. Yeast.

[b0045] Gooday G.W., Gow N.A.R. (1983). A model of the hyphal septum of *Candida albicans*. Exp. Mycol..

[b0050] Gow N.A.R., Gooday G.W., Newsam R.J., Gull K. (1980). Ultrastructure of the septum in *Candida albicans*. Curr. Microbiol..

[b0055] Gow N.A.R., Robbins P.W., Lester J.W., Brown A.J., Fonzi W.A., Chapman T., Kinsman O.S. (1994). A hyphal-specific chitin synthase gene (*CHS2*) is not essential for growth, dimorphism, or virulence of *Candida albicans*. Proc. Natl. Acad. Sci. USA.

[b0060] Jones K.H., Senft J.A. (1985). An improved method to determine cell viability by simultaneous staining with fluorescein diacetate-propidium iodide. J. Histochem. Cytochem..

[b0065] Kaloriti D., Tillmann A., Cook E., Jacobsen M., You T., Lenardon M.D., Ames L., Barahona M., Chandrasekaran K., Coghill G., Goodman D., Gow N.A.R., Grebogi C., Ho H.L., Ingram P., McDonagh A., Moura A.P., Pang W., Puttnam M., Radmaneshfar E., Romano M.C., Silk D., Stark J., Stumpf M., Thiel M., Thorne T., Usher J., Yin Z., Haynes K., Brown A.J.P. (2012). Combinatorial stresses kill pathogenic *Candida* species. Med. Mycol..

[b0070] Lenardon M.D., Milne S.A., Mora-Montes H.M., Kaffarnik F.A.R., Peck S.C., Brown A.J.P., Munro C.A., Gow N.A.R. (2010). Phosphorylation regulates polarisation of chitin synthesis in *Candida albicans*. J. Cell Sci..

[b0075] Lenardon M.D., Munro C.A., Gow N.A.R. (2010). Chitin synthesis and fungal pathogenesis. Curr. Opin. Microbiol..

[b0080] Lenardon M.D., Whitton R.K., Munro C.A., Marshall D., Gow N.A.R. (2007). Individual chitin synthase enzymes synthesize microfibrils of differing structure at specific locations in the *Candida albicans* cell wall. Mol. Microbiol..

[b0085] Mascotti K., McCullough J., Burger S.R. (2000). HPC viability measurement: trypan blue versus acridine orange and propidium iodide. Transfusion.

[b0090] Mio T., Yabe T., Sudoh M., Satoh Y., Nakajima T., Arisawa M., Yamada-Okabe H. (1996). Role of three chitin synthase genes in the growth of *Candida albicans*. J. Bacteriol..

[b0095] Motoyama T., Kojima N., Horiuchi H., Ohta A., Takagi M. (1994). Isolation of a chitin synthase gene (*chsC*) of *Aspergillus nidulans*. Biosci. Biotechnol. Biochem..

[b0100] Munro C.A., Schofield D.A., Gooday G.W., Gow N.A.R. (1998). Regulation of chitin synthesis during dimorphic growth of *Candida albicans*. Microbiology.

[b0105] Munro C.A., Selvaggini S., de Bruijn I., Walker L., Lenardon M.D., Gerssen B., Milne S., Brown A.J., Gow N.A.R. (2007). The PKC, HOG and Ca^2+^ signalling pathways co-ordinately regulate chitin synthesis in *Candida albicans*. Mol. Microbiol..

[b0110] Munro C.A., Whitton R.K., Hughes H.B., Rella M., Selvaggini S., Gow N.A.R. (2003). *CHS8*-a fourth chitin synthase gene of *Candida albicans* contributes to *in vitro* chitin synthase activity, but is dispensable for growth. Fungal Genet. Biol..

[b0115] Munro C.A., Winter K., Buchan A., Henry K., Becker J.M., Brown A.J., Bulawa C.E., Gow N.A.R. (2001). Chs1 of *Candida albicans* is an essential chitin synthase required for synthesis of the septum and for cell integrity. Mol. Microbiol..

[b0120] Noble S.M., Johnson A.D. (2005). Strains and strategies for large-scale gene deletion studies of the diploid human fungal pathogen *Candida albicans*. Eukaryot. Cell.

[b0125] Ramani R., Ramani A., Wong S.J. (1997). Rapid flow cytometric susceptibility testing of *Candida albicans*. J. Clin. Microbiol..

[b0130] Sudoh M., Yamazaki T., Masubuchi K., Taniguchi M., Shimma N., Arisawa M., Yamada-Okabe H. (2000). Identification of a novel inhibitor specific to the fungal chitin synthase. Inhibition of chitin synthase 1 arrests the cell growth, but inhibition of chitin synthase 1 and 2 is lethal in the pathogenic fungus *Candida albicans*. J. Biol. Chem..

[b0135] Veses V., Gow N.A.R. (2008). Vacuolar dynamics during the morphogenetic transition in *Candida albicans*. FEMS Yeast Res..

[b0140] Walker L.A., Lenardon M.D., Preechasuth K., Munro C.A., Gow N.A.R. (2013). Cell wall stress induces alternative fungal cytokinesis and septation strategies. J. Cell Sci..

[b0145] Walker L.A., Munro C.A., de Bruijn I., Lenardon M.D., McKinnon A., Gow N.A.R. (2008). Stimulation of chitin synthesis rescues *Candida albicans* from echinocandins. PLoS Pathog..

[b0150] Wang Z., Zheng L., Liu H., Wang Q., Hauser M., Kauffman S., Becker J.M., Szaniszlo P.J. (2001). WdChs2p, a class I chitin synthase, together with WdChs3p (class III) contributes to virulence in *Wangiella* (*Exophiala*) *dermatitidis*. Infect. Immun..

[b0155] Wilson R.B., Davis D., Enloe B.M., Mitchell A.P. (2000). A recyclable *Candida albicans URA3* cassette for PCR product-directed gene disruptions. Yeast.

[b0160] Wilson R.B., Davis D., Mitchell A.P. (1999). Rapid hypothesis testing with *Candida albicans* through gene disruption with short homology regions. J. Bacteriol..

[b0165] Wloka C., Bi E. (2012). Mechanisms of cytokinesis in budding yeast. Cytoskeleton.

