# Supplementary material for: Cell wall protection by the Candida albicans class I chitin synthases
Source: Fungal Genet Biol. 2015 Sep;82:264–76. doi: 10.1016/j.fgb.2015.08.001 (PMC4557417; doi:10.1016/j.fgb.2015.08.001)
Supplement: Supplementary Table S1 — Oligonucleotide primers used in this study. [file mmc1.docx]

**Supplementary data: Preechasuth et al.**

**Table S1: Oligonucleotide primers used in this study.**

| **Primer** | **Sequence (5’-3’)** |
| --- | --- |
| KP07 | TCTTTGTACGACCATCACTGACA |
| KP08 | AGGTTAAACAAGGATTTCATTTTTATTCCCAACTACTTTCACTTTCACTTTCACTTTCAAGGTCAGGAGTGGTTTTTATATATACAACAACTAAACAAAATTTCCCAGTCACGACGTT |
| KP09 | GACTCTAATCTCATTTCTTCTAAACTATAACAACAAAATACAATTTCTCAATATACAAATATAAATATAAATATAATTATATATATCTAAAAATATCATTTGTGGAATTGTGAGCGGATA |
| KP10 | CACTTGGACACGAAAACGAA |
| KP11 | TGACACCATGAGCATTGGTAA |
| KP12 | ATAGAGTCAGTTGCCAAGCGA |
| KP13 | TCTGATGTCGTTGTGTCGTGT |
| KP14 | CCACTCCTGACCTTGAAAGTG |
| KP15 | CGTGTGTGCAGACCAATTTGTT |
| KP16 | GATTTGGCCAACTTGAAGAGT |
| KP17 | GAGTGTTTATTTTTAGAAATCACTATATCCAACCTAGACTAAAGTTCAGTTCCCACTCATCTTTTTCACCTTTCATTATACCACTTTCATCCCTTTCGACTTTCCCAGTCACGACGTT |
| KP18 | ATACTGTATACCCCCTCCCAGATAAAACCAAATTGAAAAAAAAGAAACATTTTAATATAAATCTATATGTACAGAAAGGTCTCAAATATACACTGTTCTGTGTGGAATTGTGAGCGGATA |
| KP19 | TCGGTTTGAAGGGCAATGTA |
| KP20 | CTCTTTGTTCAATTGCCGTTG |
| KP21 | TCCGTGTGATCTGCCTAAAT |
| KP22 | GGGAAATGAAGGGGGATATAA |
| KPS222A1 | CCATTTGGAGAAGAACGAGCATTTGAAGAACCTCCTC |
| KPS222A2 | GAGGAGGTTCTTCAAATGCTCGTTCTTCTCCAAATGG |
| KPS222E1 | GCCCATTTGGAGAAGAACGAGAATTTGAAGAACCTCCTCCAC |
| KPS222E2 | GTGGAGGAGGTTCTTCAAATTCTCGTTCTTCTCCAAATGGGC |
| MDL3 | GACAGTGGCATTTATGGCATTATTTAGATTTATTGGTTGTATTTATTATTTGATCACTAGATTGGGTAGAGAAATTAAGGCAAGTGAACATGCCACTAAAGGTGGTGGTTCTAAAGGTGAAGAATTATT |
| MDL4 | TTATACTACAGGACTCTAATCTCATTTCTTCTAAACTATAACAACAAAATACAATTTCTCAATATACAAATATAAATATAAATATAATTATATATATCTATCTAGAAGGACCACCTTTGATTG |
| MDL7 | AGCATTTTTATTCTTGATATTCAAGAGTTTCCGTCCACTTAAATGGAAATTGAGAGCTAAACGAGAAAACAAACGTAATAGAAATCAAAATAGAAATGTCGGTGGTGGTTCTAAAGGTGAAGAATTATT |
| MDL8 | TATATTTCTAATACTGTATACCCCCTCCCAGATAAAACCAAATTGAAAAAAAAGAAACATTTTAATATAAATCTATATGTACAGAAAGGTCTCAAATATATCTAGAAGGACCACCTTTGATTG |
| MDL12 | GAGATAAGGCTTTGGGTTTTG |
| MDL16R | CATACCATGGGTAATACCAG |
| MDL24 | GAGTGTTTATTTTTAGAAATCACTATATCCAACCTAGACTAAAGTTCAGTTCCCACTCATCTTTTTCACCTTTCATTATACCACTTTCATCCCTTTCGACGCTCGGATCCACTAGTAACG |
| MDL25 | ATACTGTATACCCCCTCCCAGATAAAACCAAATTGAAAAAAAAGAAACATTTTAATATAAATCTATATGTACAGAAAGGTCTCAAATATACACTGTTCTGCCAGTGTGATGGATATCTGC |
| MDL28 | GTCGGTTTGAAGGGCAATG |
| MDL29 | CCATTTGGCTCTTCACTACC |
| MDL14 | GGTTTTATGTTGGGTCTGTTG |
| CHS2Ia | CATTACGCGGCCGCCTTTTTCCCTATCAGTAGTGC |
| CHS2IIb | GGCTAGTCTAGATCAAAAGAATAGTATGGGCAATAC |
| CHS2IIIa | CGTGTCGTCGACAATGATATTTTTAGATATAT |
| CHS2IIIb | AGTCACTGGGCCCTCAAAAGAATAGTATGGGCA |
