# Supplementary figures and images for: Cell wall protection by the Candida albicans class I chitin synthases
Source: Fungal Genet Biol. 2015 Sep;82:264–76. doi: 10.1016/j.fgb.2015.08.001 (PMC4557417; doi:10.1016/j.fgb.2015.08.001)

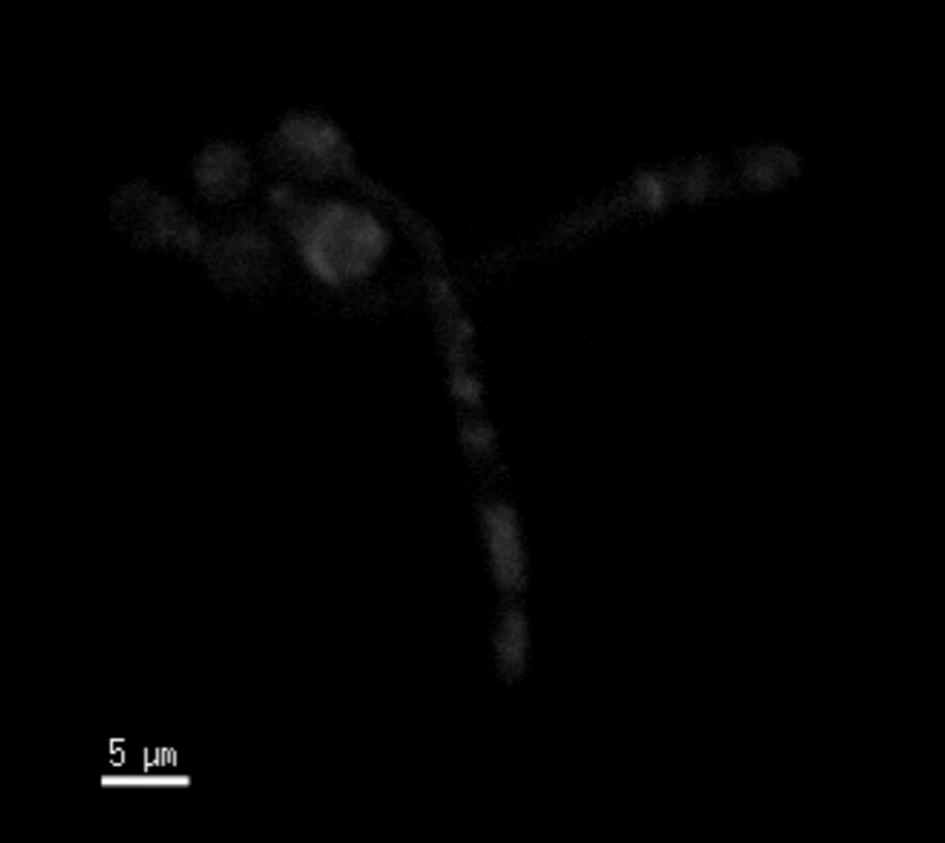

Supplement: Supplementary Movie S1 — Time-lapse movie showing the localization of Chs2-YFP in a growing hypha of the CHS2-YFP/chs2Δ0 strain. Images were recorded every 3 min for 93 min Scale bar represents 5 μm. [file mmc2.jpg]

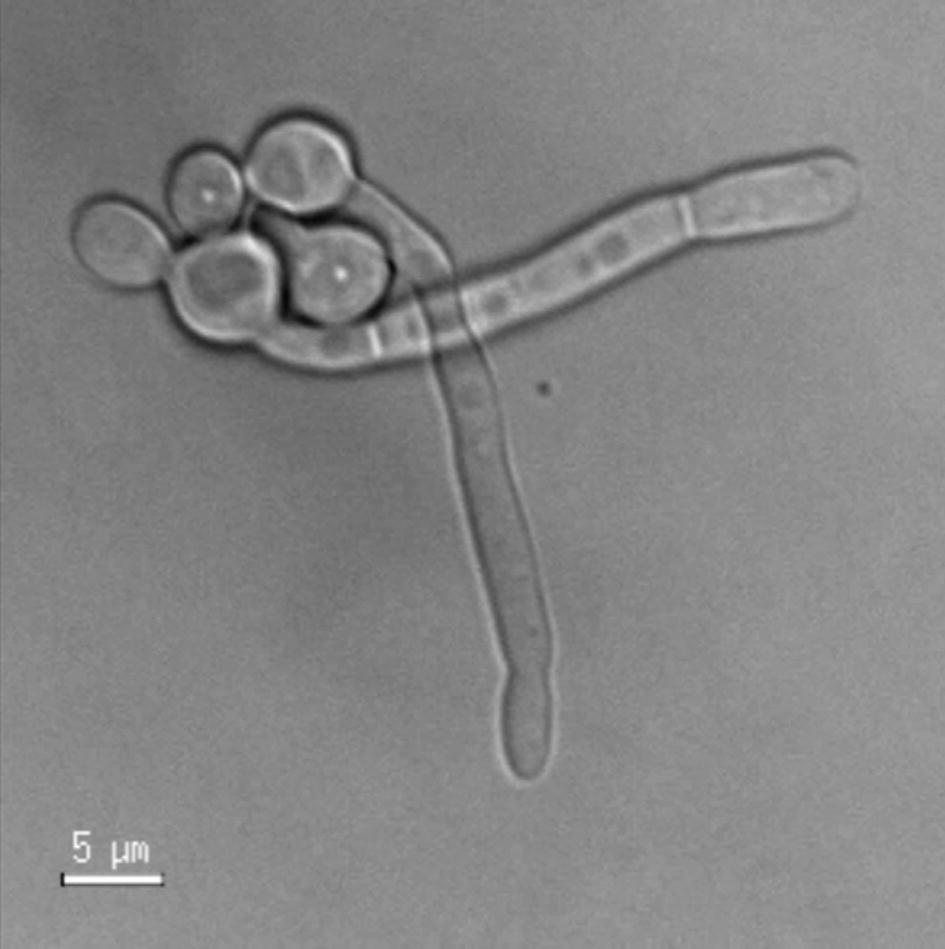

Supplement: Supplementary Movie S2 — Corresponding DIC channel of Movie S1. [file mmc3.jpg]

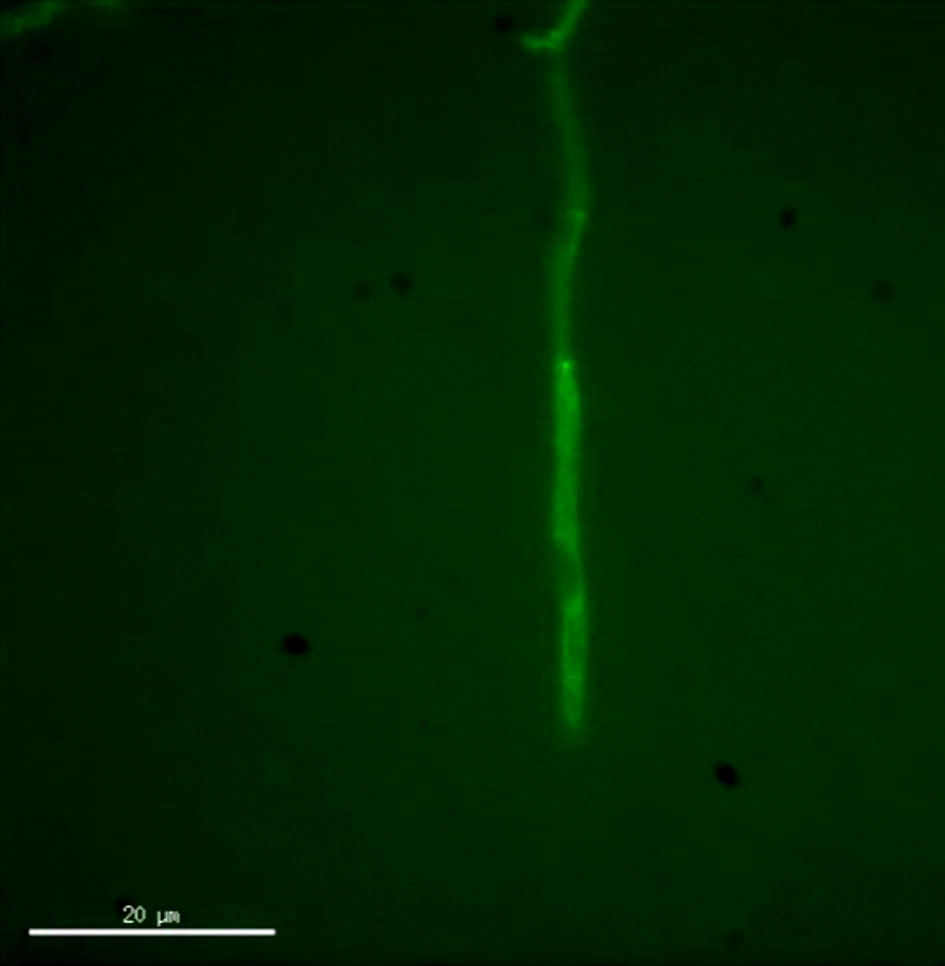

Supplement: Supplementary Movie S3 — Time-lapse movie showing the localization of Chs8-YFP in a growing hypha of the CHS8-YFP/chs8Δ0 strain. Images were recorded every 2 min for 30 min. Scale bars represent 20 μm. [file mmc4.jpg]

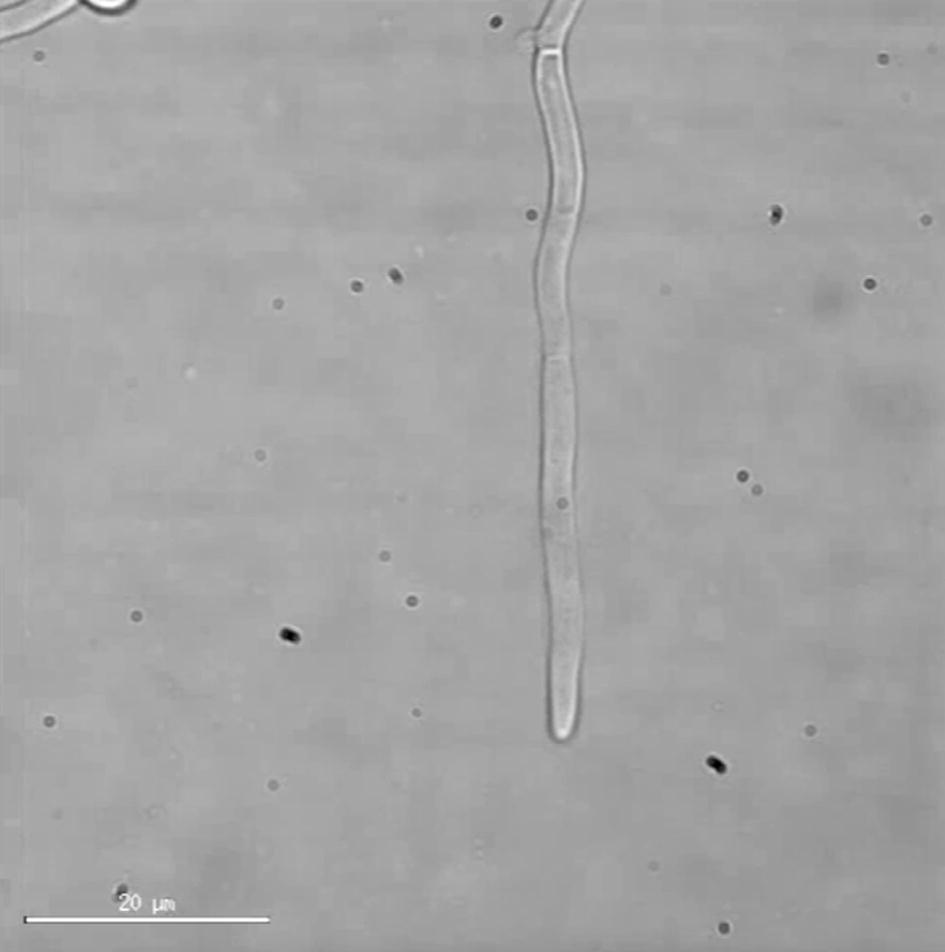

Supplement: Supplementary Movie S4 — Corresponding DIC channel of Movie S3. [file mmc5.jpg]
